# Efficient species identification for Pacific salmon genetic monitoring programs

**DOI:** 10.1111/eva.13680

**Published:** 2024-03-19

**Authors:** Zachary L. Robinson, Jeff Stephenson, Kim Vertacnik, Stuart Willis, Rebekah Horn, Jesse McCane, D. Katharine Coykendall, Shawn R. Narum

**Affiliations:** ^1^ Columbia River Inter‐Tribal Fish Commission, Hagerman Genetics Lab Hagerman Idaho USA; ^2^ Department of Entomology University of Kentucky Lexington Kentucky USA; ^3^ Eagle Fish Genetics Lab, Pacific States Marine Fisheries Commission Eagle Idaho USA

**Keywords:** amplicon sequencing, genetic monitoring, *Oncorhynchus*, Pacific salmon, species identification

## Abstract

Genetic monitoring of Pacific salmon in the Columbia River basin provides crucial information to fisheries managers that is otherwise challenging to obtain using traditional methods. Monitoring programs such as genetic stock identification (GSI) and parentage‐based tagging (PBT) involve genotyping tens of thousands of individuals annually. Although rare, these large sample collections inevitably include misidentified species, which exhibit low genotyping success on species‐specific Genotyping‐in‐Thousands by sequencing (GT‐seq) panels. For laboratories involved in large‐scale genotyping efforts, diagnosing non‐target species and reassigning them to the appropriate monitoring program can be costly and time‐consuming. To address this problem, we identified 19 primer pairs that exhibit consistent cross‐species amplification among salmonids and contain 51 species informative variants. These genetic markers reliably discriminate among 11 salmonid species and two subspecies of Cutthroat Trout and have been included in species‐specific GT‐seq panels for Chinook Salmon, Coho Salmon, Sockeye Salmon, and Rainbow Trout commonly used for Pacific salmon genetic monitoring. The majority of species‐informative amplicons (16) were newly identified from the four existing GT‐seq panels, thus demonstrating a low‐cost approach to species identification when using targeted sequencing methods. A species‐calling script was developed that is tailored for routine GT‐seq genotyping pipelines and automates the identification of non‐target species. Following extensive testing with empirical and simulated data, we demonstrated that the genetic markers and accompanying script accurately identified species and are robust to missing genotypic data and low‐frequency, shared polymorphisms among species. Finally, we used these tools to identify Coho Salmon incidentally caught in the Columbia River Chinook Salmon sport fishery and used PBT to determine their hatchery of origin. These molecular and computing resources provide a valuable tool for Pacific salmon conservation in the Columbia River basin and demonstrate a cost‐effective approach to species identification for genetic monitoring programs.

## 1 INTRODUCTION

Species misidentification is an enduring issue in fisheries science and management and occurs at all levels of expertise. A study of recreational anglers in Montana, USA, found a correct visual species identification rate of 63.0% for commonly encountered salmonid species (Schmetterling & Long, 1999). However, fisheries professionals including charter boat captains and creel surveyors have been shown to correctly identify adult salmonids at considerably higher rates than recreational anglers (Bowlby & Savoie, 2011). Nonetheless, visual species identification by trained professionals in conservation monitoring programs remains subject to human error (Tillett et al., 2012), particularly during early life‐history stages (Kirsch et al., 2018). Genetic approaches for species identification, in contrast, are highly accurate and have diverse fisheries applications including forensics and enforcement, as well as research and management (Teletchea, 2009). In certain contexts, visual species identification is difficult or impossible, and researchers must rely on genetic methods, such as identifying market substitution in processed seafood products (Cline, 2012). Given that species identification is a prerequisite for most scientific or forensic investigations, genetic approaches are now ubiquitous in the fisheries field.

Over the past few decades, DNA barcoding using highly conserved mitochondrial gene regions has become the gold standard for genetic species identification used by various federal agencies as a regulatory and enforcement tool (Bemis et al., 2023; GAO, 2009). Although many conservation‐focused genetic monitoring programs would benefit from genetic species identification with DNA barcoding, the cost of an additional workflow may be deemed prohibitive, particularly when visual misidentification is rare. However, with the rise of genotyping‐by‐sequencing it is not uncommon for genetic marker panels used for species monitoring to contain hundreds or thousands of nuclear target sequences (Meek & Larson, 2019). These target sequences may be an under‐utilized resource for addressing the issue of species misidentification in genetic monitoring collections and circumvent the need for stand‐alone genetic assays or incorporating new target sequences. For example, Beacham and Wallace (2020) identified species diagnostic variant sites for four Pacific salmon species from a single amplicon previously targeted for genetic structure analysis of Coho salmon populations (Campbell et al., 2017). In many contexts, non‐target species may also be of conservation concern and accurate species identification can provide an opportunity to gain valuable insights.

Within the Columbia River basin, large‐scale genetic monitoring programs are now an integral component of evaluating mixed‐stock fisheries and estimating stock‐specific escapement rates for wild and hatchery origin Pacific salmon (Hargrove et al., 2021; Hess et al., 2014). Effective implementation of monitoring programs that use genetic stock identification (GSI) and parentage‐based tagging (PBT) involves genotyping tens of thousands of individuals annually and requires the maintenance of large genetic baselines (Steele et al., 2019). Until recently, programs of this scale were cost‐and‐time prohibitive, but the advent of high‐throughput amplicon sequencing approaches such as Genotyping‐in‐Thousands by sequencing (GT‐seq; Campbell et al., 2015) has made these programs a practical reality. However, the sheer number of samples obtained, the often‐difficult sampling environments, and presence of multiple target species pose a variety of technical challenges, including potential species misidentification. Pacific salmon genetic monitoring programs would be improved if species‐informative markers were integrated into existing GT‐seq panels, and immediate and actionable species identification was made as part of existing bioinformatic pipelines.

The presence of non‐target species in sample collections of Pacific salmon can increase laboratory costs and result in data loss if samples are not attributed to the correct species. The GT‐seq genetic marker panels used for Columbia River basin genetic monitoring are each designed for a single species, but successful cross‐species amplification occurs and varies among markers and species (e.g., Scribner et al., 1996). A negative effect of phylogenetic distance on successful cross‐species amplification and abundance of shared polymorphisms has been empirically observed in microsatellite genetic markers (Peakall et al., 1998; Primmer et al., 2005). A similar pattern is observed in GT‐seq panels where non‐target species often fail to genotype at more loci and have lower heterozygosity than the target species for which the panel was designed (Baetscher et al., 2019; Hayward et al., 2022). Without genetic species identification, resources are often needlessly used in subsequent genotyping attempts or spent as personnel time diagnosing individual genotyping failures as generic non‐target species based on heterozygosity and genotyping success rate. In contrast, a small number of species‐informative genetic markers included in GT‐seq panels can identify non‐target species with trivial increases in costs as compared to a stand‐alone genetic assay for species identification. However, given that a variety of native and introduced salmonid fishes can be encountered in the Columbia River basin (Thurow et al., 1997; Wenger et al., 2011), it is necessary to screen for several species across three genera: *Oncorhynchus*, *Salvelinus*, and *Salmo*.

The objectives of this study were to identify species‐informative markers for salmonids potentially encountered in the Columbia River basin and incorporate a common set into four existing species‐specific GT‐seq panels used by state, federal, and tribal agencies for genetic monitoring. Instead of intensively sequencing reference samples for species marker discovery, we leverage existing GT‐seq amplicons to identify novel species‐informative variants that were unknowingly contained within well‐established genetic marker panels for Chinook Salmon (*O. tshawytscha*), Coho Salmon (*O. kisutch*), Sockeye Salmon (*O. nerka*), and Steelhead/Rainbow Trout (*O. mykiss*). After identifying and incorporating a standard set of species‐informative markers into each GT‐seq panel, we developed computing resources to automate species identification as part of existing bioinformatic pipelines. Species‐informative markers have wide‐ranging applications in conservation science and management, and leveraging existing genetic marker panels provides an efficient and cost‐effective solution to visual misidentification affecting genetic monitoring collections.

## 2 METHODS

### 2.1 Sample collections

Tissue samples for genetic analysis were obtained from eleven species and two subspecies of salmonids for initial discovery of species‐informative genetic markers. Eight species native to the Columbia River basin were included in these collections: Chinook Salmon, Coho Salmon, Sockeye Salmon, Pink Salmon (*O. gorbuscha*), Chum Salmon (*O. keta*), Steelhead/Rainbow Trout, Coastal Cutthroat Trout (*O. clarkii clarkii*), Yellowstone Cutthroat Trout (*O. clarkii bouvieri*), and Bull Trout (*Salvelinus confluentus*). Additionally, three introduced species of salmonids were included in these collections: Atlantic Salmon (*Salmo salar*), Brown Trout (*Salmo trutta*), and Brook Trout (*Salvelinus fontinalis*). Self‐sustaining populations of Brown Trout and Brook Trout are widely distributed throughout the Columbia River basin (Wenger et al., 2011). In contrast, Atlantic Salmon in Pacific drainages are largely derived from inadvertent releases from marine aquaculture with little evidence of natural reproduction (Piccolo & Orlikowska, 2012). There were 119 tissue samples used in total during the initial marker discovery, the majority of which were obtained within the Columbia River basin (Table 1). From this point forward, species abbreviations are denoted by the first letter of the genus and the first two letters of the species name (e.g., *S. trutta*: Str).

**TABLE 1:** Samples used for discovery of species‐informative variants.

Species	Source	*N*
*O. clarkii clarkii*	Hood River, OR	2
*O. clarkii bouvieri*	Upper Snake River, ID	3
*O. gorbuscha*	Coastal, WA	1
	Lower Granite Dam, WA	1
*O. keta*	Coastal, WA	1
	Lewis River, WA	1
*O. kisutch*	Makah National Fish Hatchery, WA	4
	Bonneville Fish Hatchery, OR	4
	Priest Rapids Dam, WA	4
*O. mykiss*	Skamania Hatchery, WA	8
	Big Creek Hatchery, OR	4
	Hood River, OR	4
	Yakima River, WA	4
	Upper Snake River, ID	11
	Peterson Creek, AK	1
	Alonso River, Tierra del Fuego, Chile	6
*O. nerka*	Lewis River, WA	1
	Deschutes River, OR	4
	Dworshak Reservoir, ID	4
	White River, WA	4
	Lower Granite Lake Dam, WA	1
*O. tshawytscha*	Johnson Creek, ID	4
	Nez Perce Tribal Hatchery, ID	4
	Spring Creek National Fish Hatchery, WA	4
	Yakima River, WA	4
*S. confluentus*	Warm Springs National Fish Hatchery, OR	8
*S. fontinalis*	Lago Fagnano, Tierra del Fuego, Chile	2
	Alonso River, Tierra del Fuego, Chile	1
*S. salar*	Three Commercial Sources	15
*S. trutta*	Alonso River, Tierra del Fuego, Chile	1
	Lago Fagnano, Tierra del Fuego, Chile	2
	Lago Marrow, Tierra del Fuego, Chile	1

*Note*: The source waterbody or hatchery and sample size (*N*) is provided.

Once identified, candidate species‐informative genetic markers were further validated using a distinct set of known‐species samples. Tissue samples collected throughout the Columbia River basin from Chinook Salmon (*n* = 29,677), Coho Salmon (*n* = 2205), Sockeye Salmon (*n* = 218), and Steelhead (*n* = 9755) were processed by the Hagerman Genetics Laboratory, Columbia River Inter‐Tribal Fish Commission (hereafter CRITFC) and used to test candidate species‐informative markers. These validation samples were selected using the criterion that the samples successfully genotype on their respective species‐specific GT‐seq panel at greater than 90% of polymorphic loci, which has shown to be effective as a generic filter to remove non‐target species and ensure genotyping accuracy (Hess et al., 2021; Koch & Narum, 2020). Additionally, 21 tissue samples of Coastal Cutthroat Trout were also processed on the Omy GT‐seq panel by CRITFC. Further validation testing was done by Eagle Fish Genetics Laboratory, Idaho Department of Fish and Game (hereafter EFGL) for Chinook Salmon (*n* = 8), Coho Salmon (*n* = 8), Sockeye Salmon (*n* = 8), Rainbow Trout (*n* = 8), Yellowstone Cutthroat Trout (*n* = 8), Bonneville Cutthroat Trout (*O. clarkii utah*; *n* = 8), (*n* = 16), Brook Trout (*n* = 16), and Brown Trout (*n* = 15). Notably, Bonneville Cutthroat Trout were not included during discovery of species informative variants but are more closely related to Yellowstone Cutthroat Trout compared to Coastal Cutthroat Trout (Loxterman & Keeley, 2012). Detailed descriptions of samples used during marker validation can be obtained from Table S1.

### 2.2 GT‐seq library preparation and genotyping

In this study, identical DNA extraction and GT‐seq library preparation protocols were followed for all tissue samples. DNA was extracted using Chelex100 chelating resin (Sigma‐Aldrich) following a custom protocol. GT‐seq libraries were prepared following standard protocols described in Campbell et al. (2015). Briefly, a large multiplex polymerase chain reaction (PCR) was used to amplify target sequences using species‐specific primer pools. A second PCR was used to attach Illumina adapters and dual‐index barcodes. Following PCR reactions, DNA concentrations of individual samples were normalized using Just‐a‐Plate™ (Charm BioTech) following the manufacturer's instructions and subsequently pooled. The pooled library was purified using a double‐sided size selection with magnetic beads and sequenced using 77 base pair (bp) single‐end (SE) chemistry on an Illumina NextSeq 550 or NextSeq 2000 sequencing platform (Illumina).

The GT‐seq panels and genotyping pipeline used in this study have been extensively tested (Hess et al., 2023). Prior to the addition of species‐informative markers, the species‐specific GT‐seq panels for Chinook Salmon, Coho Salmon, Sockeye Salmon, and Rainbow Trout have been widely used and described in the primary literature (e.g., Campbell et al., 2017; Collins et al., 2020; Janowitz‐Koch et al., 2019; Matala et al., 2019). The GT‐seq genetic marker panels contain 343 single‐nucleotide polymorphisms (SNPs) for Chinook Salmon, 235 SNPs for Coho Salmon, 364 SNPs for Sockeye Salmon, and 368 SNPs for Rainbow Trout. Further details including primer sequences for each of these GT‐seq panels can be found in Hess et al. (2023). Following the demultiplexing of Illumina sequence data, individual FASTQ files were genotyped using the Perl‐based scripts from Campbell et al. (2015), which were slightly modified to allow for multiple SNP variants per amplicon for this study. These updated GT‐seq genotyping scripts are freely available (https://github.com/zakrobinson/GTseq‐pipeline).

### 2.3 Discovery of species‐informative variants

Previously published and newly discovered species‐informative variants were pursued as candidates for species identification. Beacham and Wallace (2020) identified and extensively tested two highly informative amplicons, Oki‐Ots_120255 and Oki_RAD41030, each containing multiple variants for salmonid species identification. However, the primer sequences provided for Oki‐Ots_120255 produced amplicons too large to capture variants of interest with 75 bp SE Illumina sequencing chemistry tailored to existing GT‐seq panels. In response, a modified primer pair was developed for the amplicon OkiOts_120255 using Primer3 (Untergasser et al., 2012) that targets a subset of variant positions (99,105,113,115,119,133,135,137, and 141) identified by Beacham and Wallace (2020). The new primer pair for OkiOts_120255 is designated as Oki_120255mod (Table 2). The second primer pair, Oki_RAD41030, was derived from Oki_RAD41030‐31, which was originally designed to capture a Coho Salmon polymorphic variant in Campbell et al. (2017). Oki_RAD41030‐31 was already incorporated within the Coho Salmon GT‐seq panel and efficiently targets the species variants identified by Beacham and Wallace (2020) at position 51. Additionally, we used Omy_myclarp404‐111, which contains species diagnostic variant for Omy at position 45 (Narum et al., 2011), and was already incorporated into the Rainbow Trout GT‐seq panel. Importantly, these previously published variants aided in confirming the visual species identification for samples used for marker discovery.

**TABLE 2:** Description of identified amplicons containing species‐informative variants. Forward and reverse primer sequences, and variant positions within amplicon sequence are reported. The number of variants that are diagnostic for a single species (Diag.) and those that differentiate among a subset of species (Inform.) are provided for each amplicon.

Amplicon	Forward primer	Reverse primer	Variant positions	Diag.	Inform.
Oki_101419‐103^b^	CCCAATTGGAGACCAGGGTT	TCATTCAGACAGTTGGGAGACA	27,44	0	2
Oki_105105‐245^b^	GCGTATCAAGCATCAACGCC	TCTTTCAGCAAGGTTGGGCA	23	1	0
Oki_106172–60^b^	ACTACTTGGCGTGTGTGTGGG	TCCACTGAGAGGATGAGGCA	38,47,53	1	3
Oki_111681‐407^b^	TTCATCCCATTGGAAGCCCC	ACAGCCTATATCTGTGCGCT	38	1	0
Oki_120255mod^a^	GGGTAGGCTAAAACTAAATTACTCAAA	CATTGAAGGGTGGAATTGAAG	34,40,48,50,54,55,68,70,72,76	10	0
Oki_126619‐265^b^	TGCGTAGTTAATTTTCACCTCGG	TACGCAGCACTGAAGACTGG	31,35,50	2	1
Oki_aspAT‐273^b^	ATGCTGGGAGAAACAGTGGG	CTCCTCTGTAAGGGGTGGGT	37,45	1	1
Oki_RAD41030‐31^a^ ^,^ ^d^	GCTGAGCCTGGTCTGGG	TGGATACCCCAACTCCTCCA	36,37,41,55	3	1
Oki_RAD51585‐47^d^	ACTTTCTAGTAGGCGTGTGGC	CAAAACCCTGGCGTTGCAAG	25,28,31	2	1
Omy_myclarp404‐111^c^	GCTGTGGTGCTCATGGGTAAA	CCAGGGCAGGGTTGTTCTC	45	1	0
Omy_RAD13034‐67^e^ ^,^ ^f^	GAGTGATTCCCAGCCCTCC	TCTCTCCGTTGGCCAGAAAC	21,35	1	1
Omy_RAD79314‐58^e^ ^,^ ^f^	CACACTGACTCATCCCTCGC	GAGTGTCTTACCGAGCTGCC	25,27,31,66	1	3
One_1a.54542‐52^g^	GCAGGTTGTGATCGTGACCA	TGAAGAGACTACGCCCCCTT	44,47	1	1
One_2.70711‐39^g^	TGCCCTGTTGTGATGAGCAT	GGCTGTGTAGAACGACCCC	28,30,43,57,64	4	1
Ots_ARNT^h^	CCACTGGCTGTGGAGCTT	GGGTTCAGTGATAGTTGGGCAAAT	29	1	1
Ots_crRAD9615‐69^h^	GAATGCAGGGCCAGGGAG	ACTCCCAGACCATCCAGCT	19,50	1	1
Ots_myo1a‐384^h^	CTCCCCCCTGGACTTTGG	GCTCTATTGCACCGTGTTCTG	36	1	0
Ots_P53^h^	GGAACTTCCTCTCCCGTTCTG	GCACACACACGCACCTCAA	25,28,40	2	1
Ots_unk9480‐51^h^	CAAATCAGAACAAAACCTCCCACAA	GGAAGTCTGTCTGAATGGTTGTCTT	38	0	1

*Note*: Variant Positions are 1‐based positions beginning at the first base of the forward primer. To convert to nomenclature in Beacham and Wallace (2020) add 65 and 10 to the positions of Oki_120255mod and Oki_RAD41030‐31, respectively.

^a^ Beacham and Wallace (2020).

^b^ Hess et al. (2023).

^c^ Narum et al. (2011).

^d^ Campbell et al. (2017).

^e^ Chen et al. (2018).

^f^ Micheletti et al. (2018).

^g^ Matala et al. (2019).

^h^ Janowitz‐Koch et al. (2019).

Existing amplicons within species‐specific GT‐seq panels for Chinook Salmon, Coho Salmon, Sockeye Salmon, and Rainbow Trout were interrogated for novel species‐informative variants. Initial testing identified primer pairs with consistent cross‐species amplification by processing GT‐seq libraries with samples of all four species on all four species‐specific panels. Consensus sequences were then generated for each species at each candidate amplicon in *Geneious Prime* v2022.0.1 (https://www.geneious.com). These consensus sequences were aligned, and species‐informative variants were manually identified. Primer pairs that exhibited cross‐species amplification and contained species‐informative variants were incorporated into each species‐specific GT‐seq panel. With these candidate species‐informative markers incorporated, we processed GT‐seq libraries with the samples described in Table 1. The resulting amplicons were then evaluated for additional SNPs with fixed differences (i.e., *F*_ST_ = 1) among the 13 salmonid species considered. In silico allelic probes (probeSeq file; Table S2) were manually designed for each variant discovered to allow for genotyping with the GT‐seq genotyping script (Campbell et al., 2015).

### 2.4 Species scoring system

Species‐informative genetic markers do not need to be diagnostic for a single species to improve species inference. However, manually identifying the species of an unknown genetic sample based upon a series of partial inclusions and exclusions can be cumbersome, particularly in the presence of missing genotypic data. To address this issue, we developed a custom script written in Python v3.8.13 (*CallSpecies.py*; Figure S1) to automate species identification using a simple numerical scoring approach. We also tested the genetic stock identification program *Rubias* (Moran & Anderson, 2019) for species identification, but the advantages of minimizing dependencies, reformatting input files, speed of execution, and integration into existing pipeline favored our approach (Supporting Information, Appendix S1). *CallSpecies.py* uses the wide‐format, genotypic output derived from the GT‐seq genotyping pipeline (Campbell et al., 2015) and a species‐seq file that provides the locus‐specific allelic associations for each species. Using the allelic associations, species association scores are developed for each sample and the most supported species is identified (discussed in detail below). The species calling script returns the wide‐format genotypic data with two additional columns that specify the most supported species and a semicolon‐delimited string reporting the number of heterozygous and successfully genotyped species‐informative loci for each individual sample. Given that species‐informative variants with fixed differences (i.e., *F*_ST_ = 1) among species were targeted, only homozygous genotypes contribute to the species association score used to determine the species of each sample. Further, species associations can be omitted from a locus in the species‐seq file in cases where they are polymorphic or fail to genotype for a given species.

The species scoring system implemented in *CallSpecies.py* accommodates missing genotypic data and the resulting inequalities of species‐specific informativeness of remaining loci. Each row in the species‐seq file specifies the locus, allele 1 nucleotide, allele 2 nucleotide, allele 1 species associations, allele 2 species associations, and a locus‐specific scoring weight (Table S3). The species association score for all species is initialized at zero and then each species‐informative locus is evaluated for each sample. An individual sample with an allele 1 homozygous genotype for a locus will have a locus‐specific scoring weight added to all candidate species with an allele 1 association. Conversely, all species with an allele 2 association will have the locus‐specific scoring weight subtracted from their score for that individual sample (Figure S1). Missing and heterozygous genotypes do not contribute to species association scores but are counted and reported. In order for a species determination to be made, at least one candidate species must achieve a species association score that passes a user‐specified proportion of absolute maximum score threshold (‐thresMS; default: 0.5). The absolute maximum score for each species is defined by the allelic associations specified in the species‐seq file and is the maximum score that could be achieved given no missing data or heterozygous genotypes. The absolute maximum score threshold corresponds to genotype success rate for species‐informative loci when all locus‐specific scoring weights are equal. In the current study, we apply a scoring weight of two for loci that are diagnostic for a single species and a scoring weight of one for all other loci (Table S3).

In the presence of missing genotypic data for species‐informative loci, the specific combination of missing loci could result in the incorrect species achieving the highest proportion of absolute maximum score. This issue was addressed by calculating the maximum possible score achievable for each species given the missing or heterozygous genotypes observed in the sample. The species with the highest proportion of maximum possible score is ultimately called for that sample. However, multiple species are reported as a semi‐colon delimited string when their scores are within a user‐defined range of the highest observed proportion of maximum possible score (‐buffMP; default: 10^−3^). When multiple species are considered as candidates, the list of candidates can be pruned based on an additional threshold using the proportion of maximum absolute score (‐pruneMS; default: 0.34). This pruning step will remove species candidates that are supported by few loci, despite achieving a high proportion of maximum possible score. For example, a species that is described by three loci in the species‐seq file and genotypes at one locus may be retained as a candidate because it achieves a high proportion of maximum possible score based on that single locus (i.e., 1/1), but would be removed using the default pruning value based on the proportion of absolute maximum score (i.e., 1/3). Additionally, the user can specify a heterozygosity threshold for species‐informative markers after which the species call is appended with “ReviewHET” to flag potential contamination, interspecific hybridization, or shared polymorphisms among species for further review (‐thresHET; default 0.06). We use the default settings described for all analyses reported. The *CallSpecies.py* script, species‐seq file, and user manual are freely available (https://github.com/zakrobinson/CallSpecies).

### 2.5 Validation testing of species‐informative markers and scoring system

After initial discovery of species‐informative markers, they were tested using a separate set of known‐species validation samples (Table 2). The samples processed by CRITFC were genotyped on their respective, species‐specific GT‐seq panel, and the samples processed by EFGL were processed on the Chinook Salmon GT‐seq panel. Most importantly, we used these samples to determine if the identified species‐informative variants were polymorphic in any species that were expected to be monomorphic based upon initial marker discovery. Segregating variation at species‐informative variant sites reduces their value for species identification because there is no longer a single (homozygous) genotype associated with each species at each locus. This was determined by calculating allele frequencies for each variant in each species and confirming the expected homozygous species‐specific genotypic association. We then used *CallSpecies.py* to make species calls for validation samples and evaluated their concordance with visual species identification in the field. All species concordance analyses and allele frequency calculations were performed using the statistical computing software *R* v4.2.1 (R Core Team, 2022).

The performance of the species calling script and numerical scoring system was evaluated by simulating missing data and low‐frequency shared polymorphisms among species. To evaluate the effect of missing genotypic data, we simulated individual genotypes for each species based on the species associations in the species‐seq file and omitted genotypic data from a randomly selected species‐informative locus. For each species, one thousand replicate genotypes were simulated for all possible levels of missing data (i.e., {*n*_loci_, … 1}), and the affected loci were randomly selected for each simulated individual. In both the missing genotypic data and shared polymorphism simulations, the affected loci were randomly selected from loci with an expected homozygous genotype for each species (i.e., those that are informative). Therefore, the number of simulated genotypes for each species is equal to the number of informative loci multiplied by 1000 in the missing data simulation.

The effect of low‐frequency shared polymorphisms among species was tested by simulating individual genotypes for each species with a specified frequency of the alternative allele (i.e., the allele not associated with the species considered). The scenarios tested include 1, 2, 5, 10, and 15 loci effected by shared polymorphisms and a frequency of the shared polymorphisms at 0.01, 0.05, 0.1, and 0.2. Two thousand replicates were simulated for each species, alternative allele frequency, and number of affected loci. This resulted in 40,000 simulated genotypes for each species, excluding Masu Salmon (*O. masu*) because this species is diagnosed by three loci from Beacham and Wallace (2020) and only 1 and 2 affected loci were simulated (16,000 genotypes). The *CallSpecies.py* script was used to make species calls for both simulations. The concordance of called species and true species was assessed and error rates were calculated. Two types of error are reported in this investigation: (1) the proportion of species calls that fail to exclude alternative species but include the true species (true species included), and (2) the proportion of species calls that do not include the true species (true species excluded). We also report the proportion of simulated individuals for which no species determination was made. The Python scripts to conduct these simulations are distributed with *CallSpecies.py* and only require a species‐seq file as input.

### 2.6 Empirical application

In addition to discovery and validation samples used in marker development, our approach was tested on a Pacific salmon genetic monitoring collection likely to contain non‐target species. We examined 4646 putative adult Chinook Salmon tissue samples from lower Columbia River sport fishery seasons in Washington and Oregon, USA, from the years 2021 and 2022. These samples were processed using the chinook GT‐seq panel with species‐informative markers incorporated. Upon identification of non‐target species using species‐informative markers and *CallSpecies.py*, we reprocessed the samples using their corresponding species‐specific GT‐seq panel. Given that these sport fisheries are selective for hatchery origin fish, we used parentage‐based tagging (PBT) baselines of genotyped hatchery parents to assign samples that were misidentified as Chinook Salmon to their hatchery of origin. PBT baselines from Columbia River basin hatcheries can be obtained for Chinook Salmon, Coho Salmon, Sockeye Salmon, and Steelhead/Rainbow Trout from https://www.fishgen.net. Parentage assignments were made using the program *snppit* (Anderson, 2012), and were retained if the likelihood of difference score (LOD) was greater than 14 and the false discovery rate (FDR) was less than 0.1 (Hess et al., 2016).

## 3 RESULTS

### 3.1 Discovery of species‐informative variants

**FIGURE 1:**
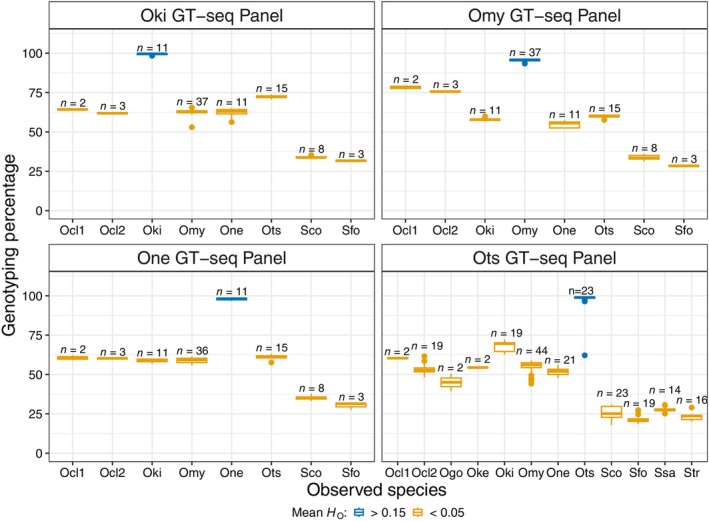
Genotyping success of known‐species samples processed on Coho Salmon, Rainbow Trout, Sockeye Salmon, and Chinook Salmon species‐specific GT‐seq panels. Each panel represents a single GT‐seq genetic marker panel. In cases where mean, observed heterozygosity of a species collection was >0.15 or <0.05 boxplots are shown in blue and orange, respectively.

Nineteen amplicons containing species‐informative variants were successfully incorporated into species‐specific GT‐seq panels for Chinook Salmon, Coho Salmon, Sockeye Salmon, and Rainbow Trout. Sixteen of these amplicons were newly identified as containing species‐informative variants and their primer pairs were obtained from among the four aforementioned GT‐seq panels. Two amplicons, Oki_120255mod and Oki_RAD41030‐31, were previously identified as species‐informative and tested in Beacham and Wallace (2020), and a third (Omy_myclarp404‐111) was identified in Narum et al. (2011). During the discovery of primer pairs on each GT‐seq panel that exhibit cross‐species amplification, the expectation was confirmed that non‐target species fail to genotype at more loci and have lower heterozygosity compared to the species for which the panel was designed (Figure 1). The primer pairs used in this investigation and their published source are provided in Table 2.

Within the identified amplicons, 51 variant positions were identified that effectively discriminate among 11 species of salmonids and two subspecies of Cutthroat Trout (Table 3). On average, each amplicon possessed 2.7 (range 1–10) SNPs (Table 2). Two amplicons, Oki_106172–60 and Ots_ARNT, contained tri‐allelic SNP variants at positions 47 and 29, respectively. These tri‐allelic variant positions received an additional entry in the species‐seq and probe‐seq files to capture the third allele within the bi‐allelic genotyping framework used in this study (Tables S2 and S3). For the variants identified that discriminate between Coastal and Yellowstone Cutthroat Trout, an ambiguous species nomenclature of Ocl1 and Ocl2 was adopted due to uncertainty of the segregation of these variants in other extant subspecies of Cutthroat Trout. For example, Bonneville Cutthroat Trout exhibited the same multi‐locus genotype as Yellowstone Cutthroat Trout using the discovered species‐informative loci and was considered part of the Ocl2 species designation. During marker discovery, one informative locus, One_2.70711–39‐43, was identified as polymorphic for Coho Salmon and, therefore, this species was omitted from species associations for this locus in the species‐seq file. The allelic associations of each species for each locus (species‐seq file) and the in silico allelic probes used to genotype these variant positions are provided (Tables S2 and S3).

**TABLE 3:** Description of species‐informative variants per species considered.

Species	Species abbrev.	Diag.	Total variants
*O. clarkii ssp*.	Ocl1	0	51
*O. clarkii ssp*.	Ocl2	3	51
*O. gorbuscha*	Ogo	6 (2)	50
*O. keta*	Oke	5 (1)	51
*O. kisutch*	Oki	4 (1)	51
*O. masu*	Oma	2 (2)	3
*O. mykiss*	Omy	2 (1)	48
*O. nerka*	One	3 (1)	51
*O. tshawytscha*	Ots	3 (1)	51
*S. confluentus*	Sco	1	44
*S. fontinalis*	Sfo	2	46
*S. Salar*	Ssa	3 (1)	43
*S. trutta*	Str	0	40

*Note*: The number of diagnostic variants for each species designation (Species Abbrev.) is reported, and of those, the numbers that are derived from Beacham and Wallace (2020) are provided in parentheses. The total number of variants that are informative and diagnostic for each species are provided.

### 3.2 Simulation testing of species scoring system

**FIGURE 2:**
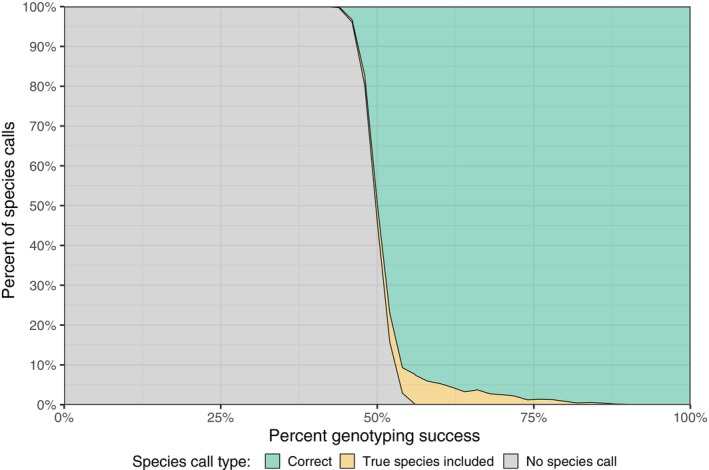
Effect of genotyping success of species‐informative loci on species calling accuracy. The proportion of simulated genotypes for each species that resulted in an exclusive and correct species call is shown in green, the proportion that included the true species along with alternative species is shown in yellow, and simulated genotypes for which no species determination was made are shown in gray. Note that no species calls were made that did not include the true species.

**FIGURE 3:**
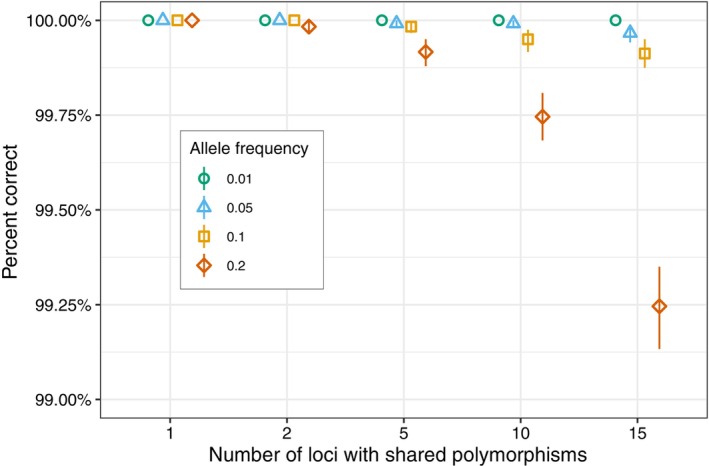
The effect of simulated shared polymorphisms among species on species calling accuracy. The percentage of species calls that are correct are reported for 1,2,5,10, and 15 loci with shared polymorphisms at an allele frequency of 0.1, 0.05, 0.1, and 0.2. The reported allele frequency corresponds to the alternate allele (i.e., the allele not associated with the species). The percentage of correct calls reflects an average among all species considered, excluding Masu Salmon. 95% bootstrapped confidence intervals are shown.

Simulation‐based testing of the species scoring system implemented in *CallSpecies.py* revealed that it was robust to missing genotypic data and low‐frequency shared polymorphisms among species. The random omission of species‐informative genotypes for each species never resulted in the true species being excluded from the species call. Considering all species, the maximum percentage of species calls that included additional species was 7.45% at a genotyping success rate of 54%–56% of species‐informative loci (Figure 2; Figure S2). In 95.05% of cases where additional species were called, only one alternative species was included with the true species, which was often the most closely related species (Table S4). In contrast, the simulation of low‐frequency shared polymorphisms among species did result in the exclusion of the true species from species calls. In the most extreme scenario tested, where 15 randomly selected loci had an alternative allele frequency of 0.2, the true species was excluded in 0.38% of species calls, averaged among all species, except Masu Salmon. However, in this scenario 99.25% of all species calls were exclusive to the true species (Figure 3). The species with the highest rate of true species exclusion (1.15%) was Brown Trout in the most extreme scenario (Figure S3–S6). For Masu Salmon, we only observed failure to make a species call when simulating shared‐polymorphisms due to default scoring thresholds and the limited number of loci for which genotypes were described (Figure S6).

### 3.3 Validation of species‐informative variants

Following discovery of species informative variants, additional testing of known‐species samples confirmed the informativeness of the identified loci. Greater than 99% of species calls using *CallSpecies.py* were correct and exclusive to a single species for all samples considered (Table 4). Importantly, only one sample of the 41,943 validation samples examined had a genetic species identification that did not match the visual identification made in the field. This error occurred in a juvenile collection of field‐identified Steelhead/Rainbow Trout at Lower Granite Dam, WA, USA, in which a specimen was called Ocl1 instead of Omy. Notably, this same collection of juveniles from Lower Granite Dam constituted 93.33% of all genetic species calls for Steelhead/Rainbow Trout in which the field‐generated species identification (Omy) was included with an alternative species (Ocl1). This result may indicate that this juvenile collection included interspecific hybrids between Cutthroat Trout and Rainbow Trout. Moreover, ambiguous genetic species identifications were not observed for returning adult, anadromous Rainbow Trout (i.e., Steelhead) collected at the same location.

**TABLE 4:** Results of species‐informative marker validation testing.

Species	Species Abbrev.^a^	Sample Source^b^	Lab^c^	*N*	Species calls			No call
					Correct (%)	True Sp. included (%)	True Sp. excluded (%)	
*O. clarkii clarkii*	Ocl1	Coquille River, OR	CRITFC	8	100.000	0.000	0.000	0
		Umpqua River, OR	CRITFC	13	100.000	0.000	0.000	0
*O. clarkii bouveri*	Ocl2	Henry's Lake, ID	EFGL	8	100.000	0.000	0.000	0
*O. clarkii utah*	Ocl2	Grace Fish Hatchery, ID	EFGL	8	100.000	0.000	0.000	0
*O. kisutch*	Oki	Columbia River basin	CRITFC	2205	99.955	0.045	0.000	0
		Nez Perce Tribal Fish Hatchery, ID	EFGL	8	100.000	0.000	0.000	0
*O. mykiss*	Omy	Columbia River basin	CRITFC	9755	99.836	0.154	0.010	0
		Lochsa River, ID	EFGL	8	100.000	0.000	0.000	0
*O. nerka*	One	Columbia River basin	CRITFC	218	99.541	0.459	0.000	0
		Eagle Fish Hatchery, ID	EFGL	8	100.000	0.000	0.000	0
*O. tshawytscha*	Ots	Columbia River basin	CRITFC	29,677	99.997	0.003	0.000	0
		Salmon River, ID	EFGL	8	100.000	0.000	0.000	0
*S. confluentus*	*Sco*	Salmon River, ID	EFGL	16	100.000	0.000	0.000	1
*S. fontinalis*	*Sfo*	Little Lost River, ID	EFGL	8	100.000	0.000	0.000	0
		Boise River, ID	EFGL	8	100.000	0.000	0.000	0
*S. trutta*	*Str*	CPW Research Hatchery, CO	EFGL	8	100.000	0.000	0.000	0

*Note*: The species, tissue sample source, processing lab, and sample size are reported. The percentage of species calls that were correct, included the true species among alternatives, and excluded the true species are provided. The number of samples for which no species call was made is also reported.

^a^ Provides the expected taxonomic grouping based upon species‐informative markers.

^b^ Detailed sampling locations are provided in Table S1.

^c^ Processing lab: Eagle Fish Genetics Lab, Eagle ID, USA (EFGL); Columbia River Inter‐Tribal Fish Commission, Hagerman, ID, USA (CRITFC).

Overall, the characterization of allele frequencies for the species informative variants confirmed their diagnostic value for species identification. Considering the nine species included in the validation samples, 92.92% of species‐specific alternative allele frequencies were estimated to be zero, and 99.53% had alternative allele frequencies of less than 0.01 (Tables S5–S13). Three variants, Oki_RAD51585‐47‐31, Oki_106172‐60‐47, and Oki_120255mod‐141, had alternative allele frequencies greater than 0.01, which were 0.038, 0.032, and 0.031 for Brown Trout, Brook Trout, and Rainbow Trout, respectively. Given the observed insensitivity of the species scoring system to low‐frequency shared polymorphisms, these loci were retained as informative for the aforementioned species. In contrast, two loci, One_2.70711‐39‐30 and One_2.70711‐39‐43, were observed during validation to have an alternative allele frequency of 0.125 for Brown Trout (Table S13), so the species association for these loci was removed from the species‐seq file. However, prior to removal of these problematic loci, the results of *CallSpecies.py* were still concordant with visual identification of Brown Trout made in the field.

### 3.4 By‐catch in Chinook Salmon sport fishery

The application of these species informative markers and the species scoring system identified low levels of Coho Salmon by‐catch in Chinook Salmon sport fishery collections. Of the 4646 putative Chinook Salmon samples examined, 486 samples failed to genotype to laboratory standards of 90% of loci on the Chinook Salmon GT‐seq panel. Concealed among these genotyping failures were 29 Coho Salmon identified by *CallSpecies.py* which successfully genotyped at 69.90% of loci on the Chinook Salmon GT‐seq panel, on average, consistent with Figure 1. Of the remaining 457 genotyping failures that were not identified as Coho Salmon, 276 were called Chinook Salmon and 181 received no species call by *CallSpecies.py*. The samples that received no species call genotyped at 9 of 51 species‐informative variants on average, and Chinook Salmon was always among the most supported species. Reprocessing the 29 putative coho samples on the Coho Salmon GT‐seq panel further confirmed their identification when they achieved high genotyping success (i.e., >90% of polymorphic loci), and were again called as Coho Salmon by the automated species scoring system. The PBT analysis identified the hatchery of origin for 10 of the 29 Coho Salmon using the PBT baseline for Columbia River basin hatchery spawn years 2012 to 2020 (dataset id:20220325; https://www.fishgen.net). The 10 Coho Salmon assigned to four different hatcheries and 9 individuals were 3+ years old and one was 2+ years old (Table S14). Excluding samples that did not receive a species call, the estimated correct visual species identification rate for this Chinook Salmon sport fishery collection was high at 99.35%.

## 4 DISCUSSION

We identified 19 amplicons that contained 51 species‐informative variants capable of distinguishing between 11 species and two subspecies of salmonid fishes. Most of the amplicons identified in this study were derived from existing GT‐seq panels for Chinook Salmon, Coho Salmon, Sockeye Salmon, and Rainbow Trout. These results demonstrate that existing amplicon sequencing assays likely contain sufficient genetic variation to discriminate among several closely related species, which provides a low‐cost solution to species misidentification in genetic monitoring collections. The numerical species scoring system implemented in *CallSpecies.py* provides a simple and automated framework for species identification within existing bioinformatic genotyping pipelines. Our empirical and simulation‐based assessments of the species scoring system and species‐informative variants demonstrated that our approach was accurate and robust to missing genotypic data and low‐frequency shared polymorphisms. The resources we provide address the need for an efficient and flexible approach to species identification for genetic monitoring of these culturally and economically important species.

Our approach of using existing amplicons to discover novel species‐informative variants has important implications for genetic monitoring using targeted sequencing assays beyond Pacific salmon. In many conservation contexts, only a few closely related species may appear as by‐catch in genetic monitoring collections. Our results suggest that reliable and accurate species identification may often be achievable solely using nuclear target sequences from established genetic monitoring panels. Merely processing known‐species samples on established targeted sequencing assays and identifying informative variants provides a low‐cost solution to species misidentification and the opportunity to use or catalog non‐target samples. In our investigation we identified SNP loci from putatively orthologous regions for the convenience of remaining within an existing genotyping framework. However, using unique haplotypes or amplicons only present in certain species is another potential strategy for genetic species identification using existing sequencing assays. Although these approaches provide a practical solution to misidentification for conservation monitoring programs, DNA barcoding using mitochondrial DNA remains the predominate method for genetic species identification in forensics and enforcement (Staats et al., 2016). It is often difficult to incorporate mitochondrial markers into amplicon sequencing assays due to the high copy number relative to nuclear markers, but this can be achieved with careful optimization (e.g., Bohling et al., 2021). Notably, the short‐read sequencing typical of GT‐seq would likely require multiple short mitochondrial haplotypes (i.e., “mini‐barcodes”; Rasmussen et al., 2009) to achieve similar resolution and accuracy to our approach.

In practice, genetic monitoring collections can be quite diverse both in phylogeographic composition and in DNA sample quality and quantity. As a result, it was important that our species scoring system was insensitive to missing genotypic data and low‐frequency shared polymorphisms. The robustness of our numerical scoring system to missing genotypic data can be attributed to the use of multiple amplicons and information from loci that are not only diagnostic to a single species but also exclude or include a subset of the species considered. Additionally, the calculation of maximum possible score per species ensured that an erroneous species call was not made because the most informative loci for any given species failed to genotype. The insensitivity of the scoring system to low‐frequency, shared polymorphisms was due to only homozygous genotypes contributing to species association scores, as heterozygous genotypes were treated as missing data. Shared polymorphisms occurring at low frequency are unlikely to be observed as homozygous for the minor allele, and thus, unlikely to affect species association scores. Similarly, minor contamination is also unlikely to result in a homozygous genotype for the minor allele. The results of our simulation suggested that the inclusion of 10 loci with minor allele frequencies of 0.05 in all species considered would have a trivial to no effect on species identification accuracy, and all scenarios examined resulted in accuracies >99.0%. Following empirical validation testing, all species‐specific minor allele frequencies were less than 0.05 and 99.6% were less than 0.01 for the retained variants. Nonetheless, the numerical scoring system we implemented is easily adapted when applied to a new system through adding or removing species or loci to the species‐seq file, and the effects of such changes can be evaluated with the distributed simulation scripts. However, some genetic population assignment software (e.g., *Rubias*; Moran & Anderson, 2019) may offer viable alternatives to our approach and achieve similar accuracies following testing and development of user‐defined assignment thresholds (Supporting Information, Appendix S1).

In our empirical example, we found that species misidentification in Chinook Salmon sport fisheries collections was rare (<1%). Without the aid of species‐informative genetic markers, this low level of Coho Salmon by‐catch would likely have been overlooked as simple failures to genotype due to poor DNA quality or laboratory errors. Our analysis of genotyping success of non‐target species shows that they often fail to genotype at >90% of loci and have lower heterozygosity than the species for which the GT‐seq panel was designed. This pattern alone is inadequate to make a reliable species identification, but combined with species‐informative markers, off‐target species within sample collections can be accurately identified. Additionally, our evaluation of genotyping success of various species on the four species‐specific GT‐seq panels revealed a general pattern of increased genotyping success for more closely related species, a pattern previously documented in cross‐species amplification studies of microsatellites (Primmer et al., 1996).

### 4.1 Caveats

Our discovery and validation samples were largely limited to the Columbia River basin, and some species were represented by few samples (Tables 1 and 2). However, the primary goal of this work was to provide a solution to species misidentification that occurs during routine genetic monitoring of Pacific salmon in the Columbia River, while creating a species identification framework that could be tested and applied to a broader geographic range. Importantly, 11 variants included in this study have been thoroughly tested in Beacham and Wallace (2020) using more broadly distributed samples of anadromous Pacific salmon species and Atlantic Salmon. These variants are diagnostic for species for which additional validation samples were not available for testing, including Pink Salmon, Chum Salmon, Masu Salmon, and Atlantic Salmon. The successful incorporation of species‐informative variants from Beacham and Wallace (2020) into these GT‐seq panels can provide additional confirmation when the novel variants identified here are applied outside the Columbia River basin.

The species scoring system we developed was not designed for identifying interspecific hybridization. However, the species calls for first‐filial (*F*1) hybrids are likely to be flagged for high heterozygosity in species‐informative markers (using default settings) and both parental species are likely to achieve nearly equivalent species association scores. This level of resolution is likely sufficient given that reproduction of *F*1 hybrids and the formation of hybrid swarms appear rare for most species in this study (Araujo et al., 2021; Devlin et al., 2022). However, introgressive hybridization is relatively common among Rainbow Trout (*O. mykiss ssp*.) and Cutthroat Trout (*O. clarkii ssp*.) subspecies in natural sympatry (Kozfkay et al., 2007) and following species introductions (Muhlfeld et al., 2014). Our approach to species identification is inadequate to identify *F*2 and later backcrosses in populations with widespread, introgressive hybridization, which would need a more intensive and directed approach.

Further complicating species identification for Cutthroat Trout is the number of extant subspecies (~12; Behnke, 2010), and ongoing taxonomic reassessment and debate (e.g., Trotter et al., 2018). In this study, our primary goal was to develop a time‐ and cost‐saving approach to make species‐level identifications for genetic monitoring collections of Pacific salmon. However, during marker discovery we identified variants that discriminate between Yellowstone and Coastal Cutthroat Trout, and during validation testing we determined that Bonneville Cutthroat Trout were genotypically consistent with Yellowstone Cutthroat Trout. As a result, we adopted the ambiguous nomenclature of Ocl1 for Coastal Cutthroat Trout, and Ocl2 for Yellowstone and Bonneville Cutthroat Trout in recognition that segregation patterns of the identified species‐informative variants in other Cutthroat Trout subspecies is unknown. Notably, this taxonomic grouping is consistent with the close phylogenetic relationship of Bonneville Cutthroat and Yellowstone Cutthroat Trout (Smith et al., 2002), and a proposed species‐level reclassification (Trotter et al., 2018). Despite these limitations, the tools provided are well‐suited for species‐level identification of Pacific salmon in the Columbia River basin and offer additional utility for Cutthroat Trout subspecies and putative *F*_1_ hybrid identification.

### 4.2 Conclusions

This work has provided a powerful suite of species‐informative markers and computing resources that enables rapid and accurate species identification for laboratories engaged in Pacific salmon genetic monitoring in the Columbia River basin. Although the resources we provide are tailored for Pacific salmon genetic monitoring, our approach of identifying variants from existing amplicon sequencing assays and the automated species scoring system can be applied to a broad array of taxa. The benefit of species calls made as part of existing bioinformatic pipelines is immense because it avoids the wasteful use of personnel and material costs diagnosing problematic (i.e., non‐target) samples. Importantly, our empirical application that identified Coho Salmon by‐catch in a chinook salmon sport fishery demonstrates that the detection of rare, non‐target species in sample collections can be of management importance and may be overlooked without resources such as those we provide here. Species identification has wide‐ranging applications in fisheries science, management, and enforcement, and this work provides molecular resources and an efficient and flexible framework for automated, genetic species identification.

## CONFLICT OF INTEREST STATEMENT

The authors declare no conflicts of interest.

## Supporting information


Data S1:

## Data Availability

Genotypic data for this study are available from the Dryad Digital Repository: https://doi.org/10.5061/dryad.nk98sf817. Python scripts associated with the manuscript are available at: https://github.com/zakrobinson/CallSpecies.
