# Supplementary material for: Efficient species identification for Pacific salmon genetic monitoring programs
Source: Evol Appl. 2024 Mar 19;17(3):e13680. doi: 10.1111/eva.13680 (PMC10950091; doi:10.1111/eva.13680)
Supplement: Supplementary file 1 — Data S1: [file EVA-17-e13680-s001.docx]

**Supplementary Material**

**ARTICLE**

**Efficient species identification for Pacific salmon genetic monitoring programs**

**Zachary L. Robinson^1^*, Jeff Stephenson^1^, Kim Vertacnik^2^, Stuart Willis^1^, Rebekah Horn^1^, Jesse McCane^3^, D. Katharine Coykendall^3^, and Shawn R. Narum^1^**

*^1^Columbia River Inter-Tribal Fish Commission, Hagerman Genetics Lab, 3059-F National Fish Hatchery Road, Hagerman, Idaho 83332, USA*

*^2^Department of Entomology, University of Kentucky, S-225 Agricultural Science Center North, Lexington, Kentucky 40546, USA*

*^3^Eagle Fish Genetics Lab, Pacific States Marine Fisheries Commission, 1800 Trout Road, Eagle, ID 83616, USA*

*Corresponding Author: *zrobinson@critfc.org*

Key words: species identification, Pacific salmon, *Oncorhynchus*, amplicon sequencing, genetic monitoring

Running title: Pacific salmon species identification

**This document includes Tables S1-S14, Figures S1 -S8, and Appendix 1.**

Table S1. Source of samples and species calling accuracy for marker validation samples. Correct = the single, true species was identified; True Sp. Included = the true species was identified along with other misidentifications; True Sp. Excluded = the true species was not identified; No Call = no species had a species association score above the threshold (see Figure S1).

|  |  |  |  |  | **Species Call** | | |  |
| --- | --- | --- | --- | --- | --- | --- | --- | --- |
| **Species** | **Species Abbrev.** | **Sample Source** | **Lab** | ***N*** | **Correct** | **True Sp. Included** | **True Sp. Excluded** | **No Call** |
| *O. clarkii clarkii* | Ocl1 | Coquille River, OR | CRITFC | 8 | 100.000% | 0.000% | 0.000% | 0 |
|  | Ocl1 | South Umpqua River, OR | CRITFC | 2 | 100.000% | 0.000% | 0.000% | 0 |
|  | Ocl1 | North Umpqua River, OR | CRITFC | 11 | 100.000% | 0.000% | 0.000% | 0 |
| *O. clarkii bouveri* | Ocl2 | Grace Fish Hatchery, ID | EFGL | 8 | 100.000% | 0.000% | 0.000% | 0 |
| *O. clarkii utah* | Ocl2 | Henry's Lake, ID | EFGL | 8 | 100.000% | 0.000% | 0.000% | 0 |
| *O. kisutch* | Oki | Mel Sampson Coho Facility, WA | CRITFC | 643 | 100.000% | 0.000% | 0.000% | 0 |
|  | Oki | Methow River, WA | CRITFC | 89 | 100.000% | 0.000% | 0.000% | 0 |
|  | Oki | Priest Rapids Dam, WA | CRITFC | 1117 | 100.000% | 0.000% | 0.000% | 0 |
|  | Oki | Wenatchee River, WA | CRITFC | 356 | 99.719% | 0.281% | 0.000% | 0 |
|  | Oki | Nez Perce Tribal Fish Hatchery, ID | EFGL | 8 | 100.000% | 0.000% | 0.000% | 0 |
| *O. mykiss* | Omy | Bonneville Dam, OR/WA | CRITFC | 30 | 100.000% | 0.000% | 0.000% | 0 |
|  | Omy | Klickitat River, WA | CRITFC | 2363 | 99.958% | 0.042% | 0.000% | 0 |
|  | Omy | Lower Granite Dam, WA | CRITFC | 6737 | 99.777% | 0.208% | 0.015% | 0 |
|  | Omy | Minthorn Springs Facility, OR | CRITFC | 37 | 100.000% | 0.000% | 0.000% | 0 |
|  | Omy | Yakima River, WA | CRITFC | 588 | 100.000% | 0.000% | 0.000% | 0 |
|  | Omy | Lochsa River, ID | EFGL | 8 | 100.000% | 0.000% | 0.000% | 0 |
| *O. nerka* | One | Bonneville Dam, OR/WA | CRITFC | 89 | 100.000% | 0.000% | 0.000% | 0 |
|  | One | Dworshak Reservoir, ID | CRITFC | 19 | 100.000% | 0.000% | 0.000% | 0 |
|  | One | Lower Columbia River, OR/WA | CRITFC | 94 | 98.936% | 1.064% | 0.000% | 0 |
|  | One | Tumwater Dam, WA | CRITFC | 16 | 100.000% | 0.000% | 0.000% | 0 |
|  | One | Eagle Fish Hatchery, ID | EFGL | 8 | 100.000% | 0.000% | 0.000% | 0 |
| *O. tshawytscha* | Ots | Bonneville Dam, OR/WA | CRITFC | 1844 | 100.000% | 0.000% | 0.000% | 0 |
|  | Ots | Chief Joseph Hatchery, WA | CRITFC | 817 | 100.000% | 0.000% | 0.000% | 0 |
|  | Ots | Cle Elum Supplementation and Research Facility, WA | CRITFC | 327 | 100.000% | 0.000% | 0.000% | 0 |
|  | Ots | Clearwater Fish Hatchery, ID | CRITFC | 2047 | 100.000% | 0.000% | 0.000% | 0 |
|  | Ots | Clearwater River, ID | CRITFC | 31 | 100.000% | 0.000% | 0.000% | 0 |
|  | Ots | Dworshak National Fish Hatchery, ID | CRITFC | 1941 | 100.000% | 0.000% | 0.000% | 0 |
|  | Ots | John Day Dam, WA | CRITFC | 347 | 100.000% | 0.000% | 0.000% | 0 |
|  | Ots | Kalama Falls Hatchery, WA | CRITFC | 138 | 100.000% | 0.000% | 0.000% | 0 |
|  | Ots | Klickitat River, WA | CRITFC | 719 | 100.000% | 0.000% | 0.000% | 0 |
|  | Ots | Little White Salmon National Fish Hatchery, WA | CRITFC | 1881 | 100.000% | 0.000% | 0.000% | 0 |
|  | Ots | Lookingglass Fish Hatchery, OR | CRITFC | 868 | 100.000% | 0.000% | 0.000% | 0 |
|  | Ots | Lower Columbia River, OR/WA | CRITFC | 3982 | 100.000% | 0.000% | 0.000% | 0 |
|  | Ots | Lower Granite Dam, WA | CRITFC | 1568 | 100.000% | 0.000% | 0.000% | 0 |
|  | Ots | Lower Snake River, WA | CRITFC | 3122 | 99.968% | 0.032% | 0.000% | 0 |
|  | Ots | Lyons Ferry Fish Hatchery, WA | CRITFC | 137 | 100.000% | 0.000% | 0.000% | 0 |
|  | Ots | Nez Perce Tribal Fish Hatchery, ID | CRITFC | 976 | 100.000% | 0.000% | 0.000% | 0 |
|  | Ots | North Santiam River, OR | CRITFC | 769 | 100.000% | 0.000% | 0.000% | 0 |
|  | Ots | Powell Satellite Fish Hatchery, ID | CRITFC | 447 | 100.000% | 0.000% | 0.000% | 0 |
|  | Ots | Priest Rapids Hatchery, WA | CRITFC | 153 | 100.000% | 0.000% | 0.000% | 0 |
|  | Ots | Rapid River Fish Hatchery, ID | CRITFC | 2261 | 100.000% | 0.000% | 0.000% | 0 |
|  | Ots | Roza Dam, WA | CRITFC | 1619 | 100.000% | 0.000% | 0.000% | 0 |
|  | Ots | Salmon River, ID | CRITFC | 1224 | 100.000% | 0.000% | 0.000% | 0 |
|  | Ots | South Santiam Fish Hatchery, OR | CRITFC | 651 | 100.000% | 0.000% | 0.000% | 0 |
|  | Ots | Speelyai Fish Hatchery, WA | CRITFC | 32 | 100.000% | 0.000% | 0.000% | 0 |
|  | Ots | Umatilla Fish Hatchery, OR | CRITFC | 67 | 100.000% | 0.000% | 0.000% | 0 |
|  | Ots | Wells Fish Hatchery, WA | CRITFC | 1514 | 100.000% | 0.000% | 0.000% | 0 |
|  | Ots | Yakima River, WA | CRITFC | 195 | 100.000% | 0.000% | 0.000% | 0 |
|  | Ots | Salmon River, ID | EFGL | 8 | 100.000% | 0.000% | 0.000% | 0 |
| *S. confluentus* | Sco | Salmon River, ID | EFGL | 16 | 100.000% | 0.000% | 0.000% | 1 |
| *S. fontinalis* | Sfo | Little Lost River, ID | EFGL | 8 | 100.000% | 0.000% | 0.000% | 0 |
|  | Sfo | Boise River, ID | EFGL | 8 | 100.000% | 0.000% | 0.000% | 0 |
| *S. trutta* | Str | CPW Research Hatchery, CO | EFGL | 15 | 100.000% | 0.000% | 0.000% | 0 |

Table S2. The *in silico* allelic probes used for genotyping via the Perl-based scripts in Campbell et al. (2015). The data are presented in probeSeq format.

| **Locus** | **A1** | **A2** | **A1-Probe** | **A2-Probe** | **FWD Primer** |
| --- | --- | --- | --- | --- | --- |
| Oki_101419-103-27 | A | C | AATCCACTTCC | AATCCCCTTCC | CCCAATTGGAGACCAGGGTT |
| Oki_101419-103-44 | T | C | GTTCTCCTACT | GTTCTCCCACT | CCCAATTGGAGACCAGGGTT |
| Oki_105105-245-23 | T | G | GCCTTTTCCAT | GCCTTGTCCAT | GCGTATCAAGCATCAACGCC |
| Oki_106172-60-38 | C | T | GTGCGCATCAA | GTGCGTATCAA | ACTACTTGGCGTGTGTGTGGG |
| Oki_106172-60-47 | G | A | AACGCGCTGAC | AACGCACTGAC | ACTACTTGGCGTGTGTGTGGG |
| Oki_106172-60-47c | A | C | AACGCACTGAC | AACGCCCTGAC | ACTACTTGGCGTGTGTGTGGG |
| Oki_106172-60-53 | C | T | CTGACCGCYGC | CTGACTGCYGC | ACTACTTGGCGTGTGTGTGGG |
| Oki_111681-407-38 | C | T | AGAAACTGCAA | AGAAACTGTAA | TTCATCCCATTGGAAGCCCC |
| Oki_120255mod-105 | C | T | AACGTCAGTTA | AACGTTAGTTA | GGGTAGGCTAAAACTAAATTACTCAAA |
| Oki_120255mod-113 | G | A | AGTTACTGG | AGTTACTAG | GGGTAGGCTAAAACTAAATTACTCAAA |
| Oki_120255mod-115 | G | T | TTACTRGGGTA | TTACTRGTGTA | GGGTAGGCTAAAACTAAATTACTCAAA |
| Oki_120255mod-119 | G | A | GTAGYTTCWTG | GTAAYTTCWTG | GGGTAGGCTAAAACTAAATTACTCAAA |
| Oki_120255mod-120 | C | T | CTTCWTGGCAT | TTTCWTGGCAT | GGGTAGGCTAAAACTAAATTACTCAAA |
| Oki_120255mod-133 | T | C | TGGCATAGTC | TGGCATAGCC | GGGTAGGCTAAAACTAAATTACTCAAA |
| Oki_120255mod-135 | A | C | GGCATAGYCAT | GGCATAGYCCT | GGGTAGGCTAAAACTAAATTACTCAAA |
| Oki_120255mod-137 | A | T | ATAGYCMTAGAT | ATAGYCMTTGAT | GGGTAGGCTAAAACTAAATTACTCAAA |
| Oki_120255mod-141 | A | G | ATAGYCMTWGATA | ATAGYCMTWGATG | GGGTAGGCTAAAACTAAATTACTCAAA |
| Oki_120255mod-99 | A | C | GACTAAAACGT | GACTACAACGT | GGGTAGGCTAAAACTAAATTACTCAAA |
| Oki_126619-265-31 | A | C | GGATTGKGCAC | GGATTGKGCCC | TGCGTAGTTAATTTTCACCTCGG |
| Oki_126619-265-35 | A | G | CAGCACAGCTG | CGGCACAGCTG | TGCGTAGTTAATTTTCACCTCGG |
| Oki_126619-265-50 | G | T | GATTCGCCAGT | GATTCTCCAGT | TGCGTAGTTAATTTTCACCTCGG |
| Oki_aspAT-273-37 | C | T | CTGGACACAAT | CTGGATACAAT | ATGCTGGGAGAAACAGTGGG |
| Oki_aspAT-273-45 | T | A | AATCCTTAACA | AATCCATAACA | ATGCTGGGAGAAACAGTGGG |
| Oki_RAD41030-31-36 | C | T | GAAGGYCRGGG | GAAGGYTRGGG | GCTGAGCCTGGTCTGGG |
| Oki_RAD41030-31-37 | G | A | GGGGSTCTGTG | AGGGSTCTGTG | GCTGAGCCTGGTCTGGG |
| Oki_RAD41030-31-41 | G | C | GGGGTCTGTGG | GGGCTCTGTGG | GCTGAGCCTGGTCTGGG |
| Oki_RAD41030-31-55 | G | T | AGCTCGGGCTG | AGCTCTGGCTG | GCTGAGCCTGGTCTGGG |
| Oki_RAD51585-47-25 | C | G | GTGGCTCACTT | GTGGCTCAGTT | ACTTTCTAGTAGGCGTGTGGC |
| Oki_RAD51585-47-28 | G | A | TCACTTGGTAGAGC | TCACTTAGTAGAGC | ACTTTCTAGTAGGCGTGTGGC |
| Oki_RAD51585-47-31 | A | C | TTKGTAGAGCA | TTKGTCGAGCA | ACTTTCTAGTAGGCGTGTGGC |
| Omy_myclarp404-111 | T | G | CAAAGCCATACGTGGCC | AAGCCATCCGTGGCC | GCTGTGGTGCTCATGGGTAAA |
| Omy_RAD13034-67-21 | C | T | CTCCCCGAACC | CTCCCTGAACC | GAGTGATTCCCAGCCCTCC |
| Omy_RAD13034-67-35 | A | T | CTGAGTGGACA | CTGTGTGGACA | GAGTGATTCCCAGCCCTCC |
| Omy_RAD79314-58-25 | C | T | CTCGCCCTGCG | CTCGCCCTGTG | CACACTGACTCATCCCTCGC |
| Omy_RAD79314-58-27 | T | C | GTCACRCCTGC | GCCACRCCTGC | CACACTGACTCATCCCTCGC |
| Omy_RAD79314-58-31 | G | A | ACGCCTGCCCT | ACACCTGCCCT | CACACTGACTCATCCCTCGC |
| Omy_RAD79314-58-66 | C | A | CAGTGTCAACC | CAGTGTCAAAC | CACACTGACTCATCCCTCGC |
| One_1a.54542-52-44 | C | T | GCCTTGTGCTT | GCCTTGTGTTT | GCAGGTTGTGATCGTGACCA |
| One_1a.54542-52-47 | G | C | TTGACCAGAAG | TTCACCAGAAG | GCAGGTTGTGATCGTGACCA |
| One_2.70711-39-28 | G | T | AGGYGCTYGTRC | ATGYGCTYGTRC | TGCCCTGTTGTGATGAGCAT |
| One_2.70711-39-30 | T | C | GTGCTYGTRCG | GCGCTYGTRCG | TGCCCTGTTGTGATGAGCAT |
| One_2.70711-39-43 | G | A | CGTCCGCGGGC | CGTCCACGGGC | TGCCCTGTTGTGATGAGCAT |
| One_2.70711-39-57 | G | A | GCTAAGGATSG | GCTAAGGATSA | TGCCCTGTTGTGATGAGCAT |
| One_2.70711-39-64 | T | G | TTCCCTAGGGG | TTCCCGAGGGG | TGCCCTGTTGTGATGAGCAT |
| Ots_ARNT-29 | A | G | GTGCTAGCTAC | GTGCTGGCTAC | CCACTGGCTGTGGAGCTT |
| Ots_ARNT-29c | A | C | GTGCTAGCTAC | GTGCTCGCTAC | CCACTGGCTGTGGAGCTT |
| Ots_crRAD9615-69-19 | T | A | GGGAGTGGGAG | GGGAGAGGGAG | GAATGCAGGGCCAGGGAG |
| Ots_crRAD9615-69-50 | A | T | CTGGGAGGAGA | CTGGGTGGAGA | GAATGCAGGGCCAGGGAG |
| Ots_myo1a-384-36 | C | T | CACCACTACCA | CACCATTACCA | CTCCCCCCTGGACTTTGG |
| Ots_P53-25 | G | T | TTCTGCAGGGG | TTCTGCAGTGG | GGAACTTCCTCTCCCGTTCTG |
| Ots_P53-28 | G | C | GGGCAGCTCCT | GGCCAGCTCCT | GGAACTTCCTCTCCCGTTCTG |
| Ots_P53-40 | T | C | CTCCTGGGTCG | CTCCTGGGCCG | GGAACTTCCTCTCCCGTTCTG |
| Ots_unk9480-51-38 | T | C | TCCCASAAACT | TCCCASAAACC | CAAATCAGAACAAAACCTCCCACAA |

Table S3. The species-seq file used in this study. Each row in the species-seq file specifies the locus, allele 1 nucleotide, allele 2 nucleotide, allele 1 species associations, allele 2 species associations, and a locus-specific scoring weight.

| **Locus** | **A1** | **A2** | **SpeciesA1** | **SpeciesA2** | **Weight** |
| --- | --- | --- | --- | --- | --- |
| Oki_101419-103-27 | A | C | Ots;Oki;Omy;Ocl1;Ocl2 | One;Ogo;Oke;Sfo;Ssa;Str | 1 |
| Oki_101419-103-44 | T | C | Ots;One;Oki;Omy;Ocl1;Ocl2;Ogo;Oke | Sfo;Ssa;Str | 1 |
| Oki_105105-245-23 | T | G | Ots;Oki;Omy;Ocl1;Ocl2;Ogo;Oke;Sfo;Sco;Ssa;Str | One | 2 |
| Oki_106172-60-38 | T | C | Ots;One;Oki;Ogo;Oke;Sfo;Sco;Ssa;Str | Omy;Ocl1;Ocl2 | 1 |
| Oki_106172-60-47 | A | G | Ots;One;Oki;Ocl2;Ogo;Oke | Omy;Ocl1 | 1 |
| Oki_106172-60-47c | A | C | Ots;One;Oki;Ocl2;Ogo;Oke | Sfo;Sco;Ssa;Str | 1 |
| Oki_106172-60-53 | T | C | Ots;One;Oki;Omy;Ocl1;Ocl2;Oke;Sfo;Sco;Ssa;Str | Ogo | 2 |
| Oki_111681-407-38 | T | C | Ots | One;Oki;Omy;Ocl1;Ocl2;Ogo;Oke;Sfo;Sco;Ssa;Str | 2 |
| Oki_120255mod-105 | C | T | Ots;One;Oki;Omy;Ocl1;Ocl2;Ogo;Oke;Sfo;Sco;Ssa;Str | Oma | 2 |
| Oki_120255mod-113 | A | G | Ots | One;Oki;Omy;Ocl1;Ocl2;Ogo;Oke;Sfo;Sco;Ssa;Str | 2 |
| Oki_120255mod-115 | G | T | Ots;Oki;Omy;Ocl1;Ocl2;Ogo;Oke;Sfo;Sco;Ssa;Str | One | 2 |
| Oki_120255mod-119 | G | A | Ots;One;Oki;Ocl1;Ocl2;Ogo;Oke;Sfo;Sco;Ssa;Str | Omy | 2 |
| Oki_120255mod-120 | C | T | Ots;One;Oki;Omy;Ocl1;Ocl2;Ogo;Oke;Sco;Ssa;Str | Sfo | 2 |
| Oki_120255mod-133 | T | C | Ots;One;Oki;Omy;Ocl1;Ocl2;Ogo;Oke;Sfo;Sco;Ssa;Str | Oma | 2 |
| Oki_120255mod-135 | A | C | Ots;One;Oki;Omy;Ocl1;Ocl2;Oke;Sfo;Sco;Ssa;Str | Ogo | 2 |
| Oki_120255mod-137 | A | T | Ots;One;Oki;Omy;Ocl1;Ocl2;Ogo;Oke;Sfo;Sco;Str | Ssa | 2 |
| Oki_120255mod-141 | A | G | Ots;One;Oki;Omy;Ocl1;Ocl2;Oke;Sfo;Sco;Ssa;Str | Ogo | 2 |
| Oki_120255mod-99 | A | C | Ots;One;Oki;Omy;Ocl1;Ocl2;Ogo;Sfo;Sco;Ssa;Str | Oke | 2 |
| Oki_126619-265-31 | A | C | Ots;One;Oki;Omy;Ocl1;Ocl2;Ogo;Oke;Sco | Sfo | 2 |
| Oki_126619-265-35 | A | G | Ots;Oki;Omy;Ocl1;Ocl2;Oke;Sfo;Sco | One;Ogo | 1 |
| Oki_126619-265-50 | G | T | Ots;One;Oki;Omy;Ocl1;Ocl2;Ogo;Sfo;Sco | Oke | 2 |
| Oki_RAD41030-31-36 | C | T | Ots;One;Oki;Ocl1;Ogo;Oke;Sfo;Sco;Ssa;Str | Ocl2 | 2 |
| Oki_RAD41030-31-37 | G | A | Ots;One;Oki;Ocl1;Ocl2;Ogo;Oke;Ssa;Str | Sfo;Sco | 1 |
| Oki_RAD41030-31-41 | G | C | Ots;Oma;One;Ocl1;Ocl2;Oke;Sfo;Sco;Ssa;Str | Oki | 2 |
| Oki_RAD41030-31-55 | G | T | Ots;One;Oki;Ocl1;Ocl2;Ogo;Oke;Sfo;Sco;Str | Ssa | 2 |
| Oki_RAD51585-47-25 | C | G | Ots;One;Oki;Omy;Ocl1;Ocl2;Ogo;Oke | Sfo;Sco;Ssa;Str | 1 |
| Oki_RAD51585-47-28 | G | A | Ots;One;Oki;Omy;Ocl1;Oke;Str | Ogo | 2 |
| Oki_RAD51585-47-31 | A | C | Ots;One;Oki;Omy;Ocl1;Ogo;Oke;Sfo;Sco;Ssa;Str | Ocl2 | 2 |
| Oki_aspAT-273-37 | C | T | Ots;One;Omy;Ocl1;Ocl2;Ogo;Oke;Sfo;Sco;Ssa;Str | Oki | 2 |
| Oki_aspAT-273-45 | T | A | Ots;One;Oki;Omy;Ocl1;Ocl2;Ogo;Oke | Sfo;Sco | 1 |
| Omy_RAD13034-67-21 | T | C | Ots | One;Oki;Omy;Ocl1;Ocl2;Ogo;Oke;Ssa | 1 |
| Omy_RAD13034-67-35 | A | T | Ots;One;Oki;Omy;Ogo;Oke;Ssa | Ocl1;Ocl2 | 2 |
| Omy_RAD79314-58-25 | C | T | Ots;Oki;Omy;Ocl1;Ocl2;Ogo;Oke;Sfo;Sco | One;Ssa;Str | 1 |
| Omy_RAD79314-58-27 | C | T | Ots;Sfo;Sco;Ssa;Str | One;Oki;Omy;Ocl1;Ocl2;Ogo;Oke | 1 |
| Omy_RAD79314-58-31 | G | A | Ots;One;Oki;Omy;Ocl1;Ocl2;Oke;Sfo;Sco;Ssa;Str | Ogo | 2 |
| Omy_RAD79314-58-66 | C | A | Ots;One;Oki;Omy;Ocl1;Ocl2;Ogo;Oke;Sfo;Sco | Ssa;Str | 1 |
| Omy_myclarp404-111 | G | T | Ots;One;Oki;Ocl1;Ocl2;Ogo;Oke;Sfo;Sco;Ssa;Str | Omy | 2 |
| One_1a.54542-52-44 | C | T | Ots;One;Oki;Omy;Ocl1;Ocl2;Ogo;Oke | Sfo;Sco;Ssa;Str | 1 |
| One_1a.54542-52-47 | G | C | Ots;One;Omy;Ocl1;Ocl2;Ogo;Oke;Sfo;Sco;Ssa;Str | Oki | 2 |
| One_2.70711-39-28 | G | T | Ots;One;Oki;Omy;Ocl1;Ocl2;Ogo;Sfo;Sco;Ssa;Str | Oke | 2 |
| One_2.70711-39-30 | T | C | Ots;One;Oki;Omy;Ocl1;Ocl2;Ogo;Oke | Sfo;Sco;Ssa | 1 |
| One_2.70711-39-43 | G | A | Ots;One;Omy;Ocl1;Ocl2;Ogo;Oke;Sfo;Sco | Ssa | 1 |
| One_2.70711-39-57 | G | A | Ots;One;Oki;Omy;Ocl1;Ocl2;Oke;Sfo;Sco;Ssa;Str | Ogo | 2 |
| One_2.70711-39-64 | T | G | Ots;One;Oki;Omy;Ocl1;Ocl2;Sfo;Sco;Ssa;Str | Oke | 2 |
| Ots_ARNT-29 | G | A | Ots;Oki;Sfo;Sco | One;Omy;Ocl1;Ocl2;Ogo | 1 |
| Ots_ARNT-29c | A | C | One;Omy;Ocl1;Ocl2;Ogo | Oke | 2 |
| Ots_P53-25 | G | T | Ots;One;Omy;Ocl1;Ocl2;Ogo;Oke;Sfo;Sco;Ssa;Str | Oki | 2 |
| Ots_P53-28 | G | C | Ots;One;Oki;Omy;Ocl1;Ocl2;Ogo;Oke;Sfo;Ssa;Str | Sco | 2 |
| Ots_P53-40 | T | C | Ots;One;Oki;Omy;Ocl1;Ocl2;Ogo;Oke | Sfo;Sco;Ssa;Str | 1 |
| Ots_crRAD9615-69-19 | T | A | Ots;Oki;Omy;Ocl1;Ocl2;Ogo;Oke;Sfo;Sco;Ssa;Str | One | 2 |
| Ots_crRAD9615-69-50 | T | A | Ots;One;Oki;Ogo;Oke | Omy;Ocl1;Ocl2;Sfo;Sco;Ssa;Str | 1 |
| Ots_myo1a-384-36 | C | T | Ots;One;Oki;Omy;Ocl1;Ogo;Oke | Ocl2 | 2 |
| Ots_unk9480-51-38 | C | T | Ots;One;Oki;Oke | Omy;Ocl1;Ocl2 | 1 |

Table S4. Results of species calls from the missing genotypic data simulation. True (simulated) species are in columns and the call made by CallSpecies.py are provided in rows. Notably, no calls exclude the true species and most multi-species calls involve a single alternative involving a closely related species (e.g., Omy,Ocl or Sco;Sfo).

|  | True Species | | | | | | | | | | | | |
| --- | --- | --- | --- | --- | --- | --- | --- | --- | --- | --- | --- | --- | --- |
| Species Call | Ocl1 | Ocl2 | Ogo | Oke | Oki | Oma | Omy | One | Ots | Sco | Sfo | Ssa | Str |
| NoCall | 25403 | 25209 | 24528 | 25496 | 25006 | 1000 | 23201 | 25668 | 25188 | 21536 | 22468 | 20988 | 19175 |
| Ocl1 | 25554 | 0 | 0 | 0 | 0 | 0 | 0 | 0 | 0 | 0 | 0 | 0 | 0 |
| Ocl1;Ocl2 | 277 | 278 | 0 | 0 | 0 | 0 | 0 | 0 | 0 | 0 | 0 | 0 | 0 |
| Ocl1;One | 1 | 0 | 0 | 0 | 0 | 0 | 0 | 0 | 0 | 0 | 0 | 0 | 0 |
| Ocl1;Sco | 3 | 0 | 0 | 0 | 0 | 0 | 0 | 0 | 0 | 2 | 0 | 0 | 0 |
| Ocl1;Sfo;Ssa;Str | 0 | 0 | 0 | 0 | 0 | 0 | 0 | 0 | 0 | 0 | 1 | 0 | 0 |
| Ocl1;Sfo;Str | 0 | 0 | 0 | 0 | 0 | 0 | 0 | 0 | 0 | 0 | 0 | 0 | 1 |
| Ocl1;Str | 8 | 0 | 0 | 0 | 0 | 0 | 0 | 0 | 0 | 0 | 0 | 0 | 6 |
| Ocl1;Str;Sco | 0 | 0 | 0 | 0 | 0 | 0 | 0 | 0 | 0 | 0 | 0 | 0 | 1 |
| Ocl2 | 0 | 26470 | 0 | 0 | 0 | 0 | 0 | 0 | 0 | 0 | 0 | 0 | 0 |
| Ogo | 0 | 0 | 25471 | 0 | 0 | 0 | 0 | 0 | 0 | 0 | 0 | 0 | 0 |
| Oke | 0 | 0 | 0 | 26502 | 0 | 0 | 0 | 0 | 0 | 0 | 0 | 0 | 0 |
| Oki | 0 | 0 | 0 | 0 | 25988 | 0 | 0 | 0 | 0 | 0 | 0 | 0 | 0 |
| Oki;Ocl1 | 1 | 0 | 0 | 0 | 0 | 0 | 0 | 0 | 0 | 0 | 0 | 0 | 0 |
| Oki;Ocl2 | 0 | 1 | 0 | 0 | 0 | 0 | 0 | 0 | 0 | 0 | 0 | 0 | 0 |
| Oki;Oke | 0 | 0 | 0 | 1 | 1 | 0 | 0 | 0 | 0 | 0 | 0 | 0 | 0 |
| Oki;Omy;Ocl1 | 0 | 0 | 0 | 0 | 1 | 0 | 0 | 0 | 0 | 0 | 0 | 0 | 0 |
| Oki;One | 0 | 0 | 0 | 0 | 1 | 0 | 0 | 0 | 0 | 0 | 0 | 0 | 0 |
| Oma | 0 | 0 | 0 | 0 | 0 | 2000 | 0 | 0 | 0 | 0 | 0 | 0 | 0 |
| Omy | 0 | 0 | 0 | 0 | 0 | 0 | 24098 | 0 | 0 | 0 | 0 | 0 | 0 |
| Omy;Ocl1 | 725 | 0 | 0 | 0 | 0 | 0 | 654 | 0 | 0 | 0 | 0 | 0 | 0 |
| Omy;Ocl1;Ocl2 | 25 | 13 | 0 | 0 | 0 | 0 | 46 | 0 | 0 | 0 | 0 | 0 | 0 |
| Omy;Ocl1;Sco | 0 | 0 | 0 | 0 | 0 | 0 | 1 | 0 | 0 | 0 | 0 | 0 | 0 |
| Omy;Ocl1;Sfo;Str | 0 | 0 | 0 | 0 | 0 | 0 | 0 | 0 | 0 | 0 | 0 | 0 | 1 |
| Omy;Ocl1;Str | 1 | 0 | 0 | 0 | 0 | 0 | 0 | 0 | 0 | 0 | 0 | 0 | 0 |
| Omy;Ocl2 | 0 | 29 | 0 | 0 | 0 | 0 | 0 | 0 | 0 | 0 | 0 | 0 | 0 |
| Omy;Sco | 0 | 0 | 0 | 0 | 0 | 0 | 0 | 0 | 0 | 1 | 0 | 0 | 0 |
| Omy;Sfo | 0 | 0 | 0 | 0 | 0 | 0 | 0 | 0 | 0 | 0 | 1 | 0 | 0 |
| One | 0 | 0 | 0 | 0 | 0 | 0 | 0 | 27331 | 0 | 0 | 0 | 0 | 0 |
| One;Sfo;Str | 0 | 0 | 0 | 0 | 0 | 0 | 0 | 0 | 0 | 0 | 0 | 0 | 1 |
| Ots | 0 | 0 | 0 | 0 | 0 | 0 | 0 | 0 | 26802 | 0 | 0 | 0 | 0 |
| Ots;Ocl1 | 2 | 0 | 0 | 0 | 0 | 0 | 0 | 0 | 1 | 0 | 0 | 0 | 0 |
| Ots;Ocl1;Ocl2;Oke;Str | 0 | 0 | 0 | 0 | 0 | 0 | 0 | 0 | 0 | 0 | 0 | 0 | 1 |
| Ots;Ogo | 0 | 0 | 1 | 0 | 0 | 0 | 0 | 0 | 0 | 0 | 0 | 0 | 0 |
| Ots;Oke | 0 | 0 | 0 | 0 | 0 | 0 | 0 | 0 | 2 | 0 | 0 | 0 | 0 |
| Ots;Oke;Str | 0 | 0 | 0 | 1 | 0 | 0 | 0 | 0 | 0 | 0 | 0 | 0 | 0 |
| Ots;Oki | 0 | 0 | 0 | 0 | 3 | 0 | 0 | 0 | 3 | 0 | 0 | 0 | 0 |
| Ots;One | 0 | 0 | 0 | 0 | 0 | 0 | 0 | 1 | 2 | 0 | 0 | 0 | 0 |
| Ots;Sco | 0 | 0 | 0 | 0 | 0 | 0 | 0 | 0 | 2 | 0 | 0 | 0 | 0 |
| Ots;Str | 0 | 0 | 0 | 0 | 0 | 0 | 0 | 0 | 0 | 0 | 0 | 0 | 1 |
| Sco | 0 | 0 | 0 | 0 | 0 | 0 | 0 | 0 | 0 | 21623 | 0 | 0 | 0 |
| Sfo | 0 | 0 | 0 | 0 | 0 | 0 | 0 | 0 | 0 | 0 | 22634 | 0 | 0 |
| Sfo;Sco | 0 | 0 | 0 | 0 | 0 | 0 | 0 | 0 | 0 | 552 | 597 | 0 | 0 |
| Sfo;Ssa;Str | 0 | 0 | 0 | 0 | 0 | 0 | 0 | 0 | 0 | 0 | 17 | 8 | 23 |
| Sfo;Ssa;Str;Sco | 0 | 0 | 0 | 0 | 0 | 0 | 0 | 0 | 0 | 1 | 1 | 12 | 8 |
| Sfo;Str | 0 | 0 | 0 | 0 | 0 | 0 | 0 | 0 | 0 | 0 | 238 | 0 | 167 |
| Sfo;Str;Sco | 0 | 0 | 0 | 0 | 0 | 0 | 0 | 0 | 0 | 44 | 43 | 0 | 80 |
| Ssa | 0 | 0 | 0 | 0 | 0 | 0 | 0 | 0 | 0 | 0 | 0 | 20342 | 0 |
| Ssa;Str | 0 | 0 | 0 | 0 | 0 | 0 | 0 | 0 | 0 | 0 | 0 | 1648 | 1590 |
| Ssa;Str;Sco | 0 | 0 | 0 | 0 | 0 | 0 | 0 | 0 | 0 | 12 | 0 | 2 | 28 |
| Str | 0 | 0 | 0 | 0 | 0 | 0 | 0 | 0 | 0 | 0 | 0 | 0 | 18786 |
| Str;Sco | 0 | 0 | 0 | 0 | 0 | 0 | 0 | 0 | 0 | 229 | 0 | 0 | 131 |

Table S5. Chinook Salmon allele frequencies for the species informative markers. Estimates are derived using validation samples from Table S3. The Exp. Allele is the allele associated with the species as defined in the species-seq file.

| **Locus** | **Species** | ***N*** | **A1** | **A2** | **Count A1** | **Count A2** | **Count Miss.** | **A1 Freq.** | **A2 Freq.** | **Miss. Freq.** | **Exp. Allele** |
| --- | --- | --- | --- | --- | --- | --- | --- | --- | --- | --- | --- |
| Omy_myclarp404-111 | Ots | 29676 | G | T | 59350 | 2 | 0 | 0.99997 | 0.00003 | 0.00000 | A1 |
| Oki_101419-103-27 | Ots | 29676 | A | C | 59352 | 0 | 0 | 1.00000 | 0.00000 | 0.00000 | A1 |
| Oki_101419-103-44 | Ots | 29676 | T | C | 59342 | 0 | 10 | 1.00000 | 0.00000 | 0.00017 | A1 |
| Oki_105105-245-23 | Ots | 29676 | T | G | 59348 | 0 | 4 | 1.00000 | 0.00000 | 0.00007 | A1 |
| Oki_106172-60-38 | Ots | 29676 | T | C | 59340 | 4 | 8 | 0.99993 | 0.00007 | 0.00013 | A1 |
| Oki_106172-60-47 | Ots | 29676 | A | G | 59341 | 3 | 8 | 0.99995 | 0.00005 | 0.00013 | A1 |
| Oki_106172-60-47c | Ots | 29676 | A | C | 59352 | 0 | 0 | 1.00000 | 0.00000 | 0.00000 | A1 |
| Oki_106172-60-53 | Ots | 29676 | T | C | 59352 | 0 | 0 | 1.00000 | 0.00000 | 0.00000 | A1 |
| Oki_111681-407-38 | Ots | 29676 | T | C | 59336 | 6 | 10 | 0.99990 | 0.00010 | 0.00017 | A1 |
| Oki_120255mod-99 | Ots | 29676 | A | C | 59350 | 0 | 2 | 1.00000 | 0.00000 | 0.00003 | A1 |
| Oki_120255mod-105 | Ots | 29676 | C | T | 59346 | 0 | 6 | 1.00000 | 0.00000 | 0.00010 | A1 |
| Oki_120255mod-113 | Ots | 29676 | A | G | 59341 | 1 | 10 | 0.99998 | 0.00002 | 0.00017 | A1 |
| Oki_120255mod-115 | Ots | 29676 | G | T | 59346 | 0 | 6 | 1.00000 | 0.00000 | 0.00010 | A1 |
| Oki_RAD41030-31-41 | Ots | 29676 | G | C | 59348 | 0 | 4 | 1.00000 | 0.00000 | 0.00007 | A1 |
| Oki_RAD51585-47-25 | Ots | 29676 | C | G | 57432 | 70 | 1850 | 0.99878 | 0.00122 | 0.03117 | A1 |
| Oki_RAD51585-47-28 | Ots | 29676 | G | A | 57610 | 0 | 1742 | 1.00000 | 0.00000 | 0.02935 | A1 |
| Oki_RAD51585-47-31 | Ots | 29676 | A | C | 57816 | 0 | 1536 | 1.00000 | 0.00000 | 0.02588 | A1 |
| Omy_RAD13034-67-21 | Ots | 29676 | T | C | 59343 | 5 | 4 | 0.99992 | 0.00008 | 0.00007 | A1 |
| Omy_RAD13034-67-35 | Ots | 29676 | A | T | 59336 | 0 | 16 | 1.00000 | 0.00000 | 0.00027 | A1 |
| Oki_120255mod-119 | Ots | 29676 | G | A | 59346 | 0 | 6 | 1.00000 | 0.00000 | 0.00010 | A1 |
| Oki_120255mod-120 | Ots | 29676 | C | T | 59350 | 0 | 2 | 1.00000 | 0.00000 | 0.00003 | A1 |
| Oki_120255mod-133 | Ots | 29676 | T | C | 59350 | 0 | 2 | 1.00000 | 0.00000 | 0.00003 | A1 |
| Oki_120255mod-135 | Ots | 29676 | A | C | 59350 | 0 | 2 | 1.00000 | 0.00000 | 0.00003 | A1 |
| Oki_120255mod-137 | Ots | 29676 | A | T | 59350 | 0 | 2 | 1.00000 | 0.00000 | 0.00003 | A1 |
| Oki_120255mod-141 | Ots | 29676 | A | G | 59350 | 0 | 2 | 1.00000 | 0.00000 | 0.00003 | A1 |
| Oki_126619-265-31 | Ots | 29676 | A | C | 59352 | 0 | 0 | 1.00000 | 0.00000 | 0.00000 | A1 |
| Oki_126619-265-35 | Ots | 29676 | A | G | 59350 | 0 | 2 | 1.00000 | 0.00000 | 0.00003 | A1 |
| Oki_126619-265-50 | Ots | 29676 | G | T | 59352 | 0 | 0 | 1.00000 | 0.00000 | 0.00000 | A1 |
| Oki_aspAT-273-37 | Ots | 29676 | C | T | 59350 | 0 | 2 | 1.00000 | 0.00000 | 0.00003 | A1 |
| Oki_aspAT-273-45 | Ots | 29676 | T | A | 59352 | 0 | 0 | 1.00000 | 0.00000 | 0.00000 | A1 |
| Oki_RAD41030-31-36 | Ots | 29676 | C | T | 59335 | 17 | 0 | 0.99971 | 0.00029 | 0.00000 | A1 |
| Oki_RAD41030-31-37 | Ots | 29676 | G | A | 59315 | 35 | 2 | 0.99941 | 0.00059 | 0.00003 | A1 |
| Omy_RAD79314-58-25 | Ots | 29676 | C | T | 59352 | 0 | 0 | 1.00000 | 0.00000 | 0.00000 | A1 |
| Omy_RAD79314-58-27 | Ots | 29676 | C | T | 59343 | 3 | 6 | 0.99995 | 0.00005 | 0.00010 | A1 |
| Omy_RAD79314-58-31 | Ots | 29676 | G | A | 59352 | 0 | 0 | 1.00000 | 0.00000 | 0.00000 | A1 |
| Omy_RAD79314-58-66 | Ots | 29676 | C | A | 59352 | 0 | 0 | 1.00000 | 0.00000 | 0.00000 | A1 |
| One_1a.54542-52-44 | Ots | 29676 | C | T | 59352 | 0 | 0 | 1.00000 | 0.00000 | 0.00000 | A1 |
| One_1a.54542-52-47 | Ots | 29676 | G | C | 59351 | 1 | 0 | 0.99998 | 0.00002 | 0.00000 | A1 |
| One_2.70711-39-28 | Ots | 29676 | G | T | 59352 | 0 | 0 | 1.00000 | 0.00000 | 0.00000 | A1 |
| One_2.70711-39-30 | Ots | 29676 | T | C | 59352 | 0 | 0 | 1.00000 | 0.00000 | 0.00000 | A1 |
| One_2.70711-39-43 | Ots | 29676 | G | A | 59352 | 0 | 0 | 1.00000 | 0.00000 | 0.00000 | A1 |
| One_2.70711-39-57 | Ots | 29676 | G | A | 59352 | 0 | 0 | 1.00000 | 0.00000 | 0.00000 | A1 |
| One_2.70711-39-64 | Ots | 29676 | T | G | 59352 | 0 | 0 | 1.00000 | 0.00000 | 0.00000 | A1 |
| Ots_ARNT-29 | Ots | 29676 | G | A | 59352 | 0 | 0 | 1.00000 | 0.00000 | 0.00000 | A1 |
| Ots_ARNT-29c | Ots | 29676 | A | C | 44 | 0 | 59308 | 1.00000 | 0.00000 | 0.99926 | – |
| Ots_crRAD9615-69-19 | Ots | 29676 | T | A | 59336 | 0 | 16 | 1.00000 | 0.00000 | 0.00027 | A1 |
| Ots_crRAD9615-69-50 | Ots | 29676 | T | A | 59342 | 2 | 8 | 0.99997 | 0.00003 | 0.00013 | A1 |
| Ots_myo1a-384-36 | Ots | 29676 | C | T | 59344 | 0 | 8 | 1.00000 | 0.00000 | 0.00013 | A1 |
| Ots_P53-25 | Ots | 29676 | G | T | 59352 | 0 | 0 | 1.00000 | 0.00000 | 0.00000 | A1 |
| Ots_P53-28 | Ots | 29676 | G | C | 59352 | 0 | 0 | 1.00000 | 0.00000 | 0.00000 | A1 |
| Ots_P53-40 | Ots | 29676 | T | C | 59352 | 0 | 0 | 1.00000 | 0.00000 | 0.00000 | A1 |
| Ots_unk9480-51-38 | Ots | 29676 | C | T | 59340 | 4 | 8 | 0.99993 | 0.00007 | 0.00013 | A1 |
| Oki_RAD41030-31-55 | Ots | 29676 | G | T | 32478 | 0 | 26874 | 1.00000 | 0.00000 | 0.45279 | A1 |

Table S6. Coho Salmon allele frequencies for the species informative markers. Estimates are derived using validation samples from Table S3. The Exp. Allele is the allele associated with the species as defined in the species-seq file.

| **Locus** | **Species** | ***N*** | **A1** | **A2** | **Count A1** | **Count A2** | **Count Miss.** | **A1 Freq.** | **A2 Freq.** | **Miss. Freq.** | **Exp. Allele** |
| --- | --- | --- | --- | --- | --- | --- | --- | --- | --- | --- | --- |
| Oki_101419-103-27 | Oki | 2205 | A | C | 4410 | 0 | 0 | 1.0000 | 0.0000 | 0.0000 | A1 |
| Oki_101419-103-44 | Oki | 2205 | T | C | 4410 | 0 | 0 | 1.0000 | 0.0000 | 0.0000 | A1 |
| Oki_105105-245-23 | Oki | 2205 | T | G | 4410 | 0 | 0 | 1.0000 | 0.0000 | 0.0000 | A1 |
| Oki_106172-60-38 | Oki | 2205 | T | C | 4396 | 4 | 10 | 0.9991 | 0.0009 | 0.0023 | A1 |
| Oki_106172-60-47 | Oki | 2205 | A | G | 4396 | 4 | 10 | 0.9991 | 0.0009 | 0.0023 | A1 |
| Oki_106172-60-47c | Oki | 2205 | A | C | 4408 | 0 | 2 | 1.0000 | 0.0000 | 0.0005 | A1 |
| Oki_106172-60-53 | Oki | 2205 | T | C | 4408 | 0 | 2 | 1.0000 | 0.0000 | 0.0005 | A1 |
| Oki_111681-407-38 | Oki | 2205 | T | C | 15 | 4373 | 22 | 0.0034 | 0.9966 | 0.0050 | A2 |
| Oki_120255mod-105 | Oki | 2205 | C | T | 4394 | 0 | 16 | 1.0000 | 0.0000 | 0.0036 | A1 |
| Oki_120255mod-113 | Oki | 2205 | A | G | 1 | 4373 | 36 | 0.0002 | 0.9998 | 0.0082 | A2 |
| Oki_120255mod-115 | Oki | 2205 | G | T | 4394 | 0 | 16 | 1.0000 | 0.0000 | 0.0036 | A1 |
| Oki_120255mod-119 | Oki | 2205 | G | A | 4392 | 0 | 18 | 1.0000 | 0.0000 | 0.0041 | A1 |
| Oki_120255mod-120 | Oki | 2205 | C | T | 4392 | 0 | 18 | 1.0000 | 0.0000 | 0.0041 | A1 |
| Oki_120255mod-133 | Oki | 2205 | T | C | 4392 | 0 | 18 | 1.0000 | 0.0000 | 0.0041 | A1 |
| Oki_120255mod-135 | Oki | 2205 | A | C | 4394 | 0 | 16 | 1.0000 | 0.0000 | 0.0036 | A1 |
| Oki_120255mod-137 | Oki | 2205 | A | T | 4392 | 0 | 18 | 1.0000 | 0.0000 | 0.0041 | A1 |
| Oki_120255mod-141 | Oki | 2205 | A | G | 4392 | 0 | 18 | 1.0000 | 0.0000 | 0.0041 | A1 |
| Oki_120255mod-99 | Oki | 2205 | A | C | 4394 | 0 | 16 | 1.0000 | 0.0000 | 0.0036 | A1 |
| Oki_126619-265-31 | Oki | 2205 | A | C | 4410 | 0 | 0 | 1.0000 | 0.0000 | 0.0000 | A1 |
| Oki_126619-265-35 | Oki | 2205 | A | G | 4410 | 0 | 0 | 1.0000 | 0.0000 | 0.0000 | A1 |
| Oki_126619-265-50 | Oki | 2205 | G | T | 4410 | 0 | 0 | 1.0000 | 0.0000 | 0.0000 | A1 |
| Oki_aspAT-273-37 | Oki | 2205 | C | T | 17 | 4365 | 28 | 0.0039 | 0.9961 | 0.0063 | A2 |
| Oki_aspAT-273-45 | Oki | 2205 | T | A | 4410 | 0 | 0 | 1.0000 | 0.0000 | 0.0000 | A1 |
| Oki_RAD41030-31-36 | Oki | 2205 | C | T | 4410 | 0 | 0 | 1.0000 | 0.0000 | 0.0000 | A1 |
| Oki_RAD41030-31-37 | Oki | 2205 | G | A | 4410 | 0 | 0 | 1.0000 | 0.0000 | 0.0000 | A1 |
| Oki_RAD41030-31-41 | Oki | 2205 | G | C | 4 | 4380 | 26 | 0.0009 | 0.9991 | 0.0059 | A2 |
| Oki_RAD41030-31-55 | Oki | 2205 | G | T | 3124 | 0 | 1286 | 1.0000 | 0.0000 | 0.2916 | A1 |
| Oki_RAD51585-47-25 | Oki | 2205 | C | G | 4410 | 0 | 0 | 1.0000 | 0.0000 | 0.0000 | A1 |
| Oki_RAD51585-47-28 | Oki | 2205 | G | A | 3402 | 0 | 1008 | 1.0000 | 0.0000 | 0.2286 | A1 |
| Oki_RAD51585-47-31 | Oki | 2205 | A | C | 4410 | 0 | 0 | 1.0000 | 0.0000 | 0.0000 | A1 |
| Omy_myclarp404-111 | Oki | 2205 | G | T | 4402 | 4 | 4 | 0.9991 | 0.0009 | 0.0009 | A1 |
| Omy_RAD13034-67-21 | Oki | 2205 | T | C | 3 | 4397 | 10 | 0.0007 | 0.9993 | 0.0023 | A2 |
| Omy_RAD13034-67-35 | Oki | 2205 | A | T | 4410 | 0 | 0 | 1.0000 | 0.0000 | 0.0000 | A1 |
| Omy_RAD79314-58-25 | Oki | 2205 | C | T | 4410 | 0 | 0 | 1.0000 | 0.0000 | 0.0000 | A1 |
| Omy_RAD79314-58-27 | Oki | 2205 | C | T | 7 | 4345 | 58 | 0.0016 | 0.9984 | 0.0132 | A2 |
| Omy_RAD79314-58-31 | Oki | 2205 | G | A | 4410 | 0 | 0 | 1.0000 | 0.0000 | 0.0000 | A1 |
| Omy_RAD79314-58-66 | Oki | 2205 | C | A | 4410 | 0 | 0 | 1.0000 | 0.0000 | 0.0000 | A1 |
| One_1a.54542-52-44 | Oki | 2205 | C | T | 4410 | 0 | 0 | 1.0000 | 0.0000 | 0.0000 | A1 |
| One_1a.54542-52-47 | Oki | 2205 | G | C | 15 | 4359 | 36 | 0.0034 | 0.9966 | 0.0082 | A2 |
| One_2.70711-39-28 | Oki | 2205 | G | T | 4410 | 0 | 0 | 1.0000 | 0.0000 | 0.0000 | A1 |
| One_2.70711-39-30 | Oki | 2205 | T | C | 4410 | 0 | 0 | 1.0000 | 0.0000 | 0.0000 | A1 |
| One_2.70711-39-43 | Oki | 2205 | G | A | 858 | 3474 | 78 | 0.1981 | 0.8019 | 0.0177 | – |
| One_2.70711-39-57 | Oki | 2205 | G | A | 4410 | 0 | 0 | 1.0000 | 0.0000 | 0.0000 | A1 |
| One_2.70711-39-64 | Oki | 2205 | T | G | 4410 | 0 | 0 | 1.0000 | 0.0000 | 0.0000 | A1 |
| Ots_ARNT-29 | Oki | 2205 | G | A | 4409 | 1 | 0 | 0.9998 | 0.0002 | 0.0000 | A1 |
| Ots_ARNT-29c | Oki | 2205 | A | C | 22 | 0 | 4388 | 1.0000 | 0.0000 | 0.9950 | – |
| Ots_crRAD9615-69-19 | Oki | 2205 | T | A | 4394 | 0 | 16 | 1.0000 | 0.0000 | 0.0036 | A1 |
| Ots_crRAD9615-69-50 | Oki | 2205 | T | A | 4390 | 4 | 16 | 0.9991 | 0.0009 | 0.0036 | A1 |
| Ots_myo1a-384-36 | Oki | 2205 | C | T | 4410 | 0 | 0 | 1.0000 | 0.0000 | 0.0000 | A1 |
| Ots_P53-25 | Oki | 2205 | G | T | 17 | 4369 | 24 | 0.0039 | 0.9961 | 0.0054 | A2 |
| Ots_P53-28 | Oki | 2205 | G | C | 4410 | 0 | 0 | 1.0000 | 0.0000 | 0.0000 | A1 |
| Ots_P53-40 | Oki | 2205 | T | C | 4410 | 0 | 0 | 1.0000 | 0.0000 | 0.0000 | A1 |
| Ots_unk9480-51-38 | Oki | 2205 | C | T | 4392 | 4 | 14 | 0.9991 | 0.0009 | 0.0032 | A1 |

Table S7. Sockeye Salmon allele frequencies for the species informative markers. Estimates are derived using validation samples from Table S3. The Exp. Allele is the allele associated with the species as defined in the species-seq file.

| **Locus** | **Species** | ***N*** | **A1** | **A2** | **Count A1** | **Count A2** | **Count Miss.** | **A1 Freq.** | **A2 Freq.** | **Miss. Freq.** | **Exp. Allele** |
| --- | --- | --- | --- | --- | --- | --- | --- | --- | --- | --- | --- |
| Oki_101419-103-27 | One | 218 | A | C | 1 | 435 | 0 | 0.002 | 0.998 | 0.000 | A2 |
| Oki_101419-103-44 | One | 218 | T | C | 436 | 0 | 0 | 1.000 | 0.000 | 0.000 | A1 |
| Oki_105105-245-23 | One | 218 | T | G | 0 | 434 | 2 | 0.000 | 1.000 | 0.005 | A2 |
| Oki_106172-60-38 | One | 218 | T | C | 436 | 0 | 0 | 1.000 | 0.000 | 0.000 | A1 |
| Oki_106172-60-47 | One | 218 | A | G | 436 | 0 | 0 | 1.000 | 0.000 | 0.000 | A1 |
| Oki_106172-60-47c | One | 218 | A | C | 436 | 0 | 0 | 1.000 | 0.000 | 0.000 | A1 |
| Oki_106172-60-53 | One | 218 | T | C | 436 | 0 | 0 | 1.000 | 0.000 | 0.000 | A1 |
| Oki_111681-407-38 | One | 218 | T | C | 0 | 434 | 2 | 0.000 | 1.000 | 0.005 | A2 |
| Oki_120255mod-105 | One | 218 | C | T | 434 | 0 | 2 | 1.000 | 0.000 | 0.005 | A1 |
| Oki_120255mod-113 | One | 218 | A | G | 0 | 424 | 12 | 0.000 | 1.000 | 0.028 | A2 |
| Oki_120255mod-115 | One | 218 | G | T | 0 | 422 | 14 | 0.000 | 1.000 | 0.032 | A2 |
| Oki_120255mod-119 | One | 218 | G | A | 434 | 0 | 2 | 1.000 | 0.000 | 0.005 | A1 |
| Oki_120255mod-120 | One | 218 | C | T | 434 | 0 | 2 | 1.000 | 0.000 | 0.005 | A1 |
| Oki_120255mod-133 | One | 218 | T | C | 434 | 0 | 2 | 1.000 | 0.000 | 0.005 | A1 |
| Oki_120255mod-135 | One | 218 | A | C | 434 | 0 | 2 | 1.000 | 0.000 | 0.005 | A1 |
| Oki_120255mod-137 | One | 218 | A | T | 434 | 0 | 2 | 1.000 | 0.000 | 0.005 | A1 |
| Oki_120255mod-141 | One | 218 | A | G | 434 | 0 | 2 | 1.000 | 0.000 | 0.005 | A1 |
| Oki_120255mod-99 | One | 218 | A | C | 434 | 0 | 2 | 1.000 | 0.000 | 0.005 | A1 |
| Oki_126619-265-31 | One | 218 | A | C | 436 | 0 | 0 | 1.000 | 0.000 | 0.000 | A1 |
| Oki_126619-265-35 | One | 218 | A | G | 0 | 432 | 4 | 0.000 | 1.000 | 0.009 | A2 |
| Oki_126619-265-50 | One | 218 | G | T | 436 | 0 | 0 | 1.000 | 0.000 | 0.000 | A1 |
| Oki_aspAT-273-37 | One | 218 | C | T | 434 | 0 | 2 | 1.000 | 0.000 | 0.005 | A1 |
| Oki_aspAT-273-45 | One | 218 | T | A | 436 | 0 | 0 | 1.000 | 0.000 | 0.000 | A1 |
| Oki_RAD41030-31-36 | One | 218 | C | T | 436 | 0 | 0 | 1.000 | 0.000 | 0.000 | A1 |
| Oki_RAD41030-31-37 | One | 218 | G | A | 436 | 0 | 0 | 1.000 | 0.000 | 0.000 | A1 |
| Oki_RAD41030-31-41 | One | 218 | G | C | 434 | 0 | 2 | 1.000 | 0.000 | 0.005 | A1 |
| Oki_RAD41030-31-55 | One | 218 | G | T | 436 | 0 | 0 | 1.000 | 0.000 | 0.000 | A1 |
| Oki_RAD51585-47-25 | One | 218 | C | G | 358 | 0 | 78 | 1.000 | 0.000 | 0.179 | A1 |
| Oki_RAD51585-47-28 | One | 218 | G | A | 358 | 0 | 78 | 1.000 | 0.000 | 0.179 | A1 |
| Oki_RAD51585-47-31 | One | 218 | A | C | 360 | 0 | 76 | 1.000 | 0.000 | 0.174 | A1 |
| Omy_myclarp404-111 | One | 218 | G | T | 436 | 0 | 0 | 1.000 | 0.000 | 0.000 | A1 |
| Omy_RAD13034-67-21 | One | 218 | T | C | 0 | 436 | 0 | 0.000 | 1.000 | 0.000 | A2 |
| Omy_RAD13034-67-35 | One | 218 | A | T | 436 | 0 | 0 | 1.000 | 0.000 | 0.000 | A1 |
| Omy_RAD79314-58-25 | One | 218 | C | T | 0 | 432 | 4 | 0.000 | 1.000 | 0.009 | A2 |
| Omy_RAD79314-58-27 | One | 218 | C | T | 0 | 436 | 0 | 0.000 | 1.000 | 0.000 | A2 |
| Omy_RAD79314-58-31 | One | 218 | G | A | 436 | 0 | 0 | 1.000 | 0.000 | 0.000 | A1 |
| Omy_RAD79314-58-66 | One | 218 | C | A | 436 | 0 | 0 | 1.000 | 0.000 | 0.000 | A1 |
| One_1a.54542-52-44 | One | 218 | C | T | 436 | 0 | 0 | 1.000 | 0.000 | 0.000 | A1 |
| One_1a.54542-52-47 | One | 218 | G | C | 434 | 0 | 2 | 1.000 | 0.000 | 0.005 | A1 |
| One_2.70711-39-28 | One | 218 | G | T | 436 | 0 | 0 | 1.000 | 0.000 | 0.000 | A1 |
| One_2.70711-39-30 | One | 218 | T | C | 436 | 0 | 0 | 1.000 | 0.000 | 0.000 | A1 |
| One_2.70711-39-43 | One | 218 | G | A | 434 | 0 | 2 | 1.000 | 0.000 | 0.005 | A1 |
| One_2.70711-39-57 | One | 218 | G | A | 436 | 0 | 0 | 1.000 | 0.000 | 0.000 | A1 |
| One_2.70711-39-64 | One | 218 | T | G | 436 | 0 | 0 | 1.000 | 0.000 | 0.000 | A1 |
| Ots_ARNT-29 | One | 218 | G | A | 0 | 434 | 2 | 0.000 | 1.000 | 0.005 | A2 |
| Ots_ARNT-29c | One | 218 | A | C | 436 | 0 | 0 | 1.000 | 0.000 | 0.000 | A1 |
| Ots_crRAD9615-69-19 | One | 218 | T | A | 0 | 432 | 4 | 0.000 | 1.000 | 0.009 | A2 |
| Ots_crRAD9615-69-50 | One | 218 | T | A | 436 | 0 | 0 | 1.000 | 0.000 | 0.000 | A1 |
| Ots_myo1a-384-36 | One | 218 | C | T | 436 | 0 | 0 | 1.000 | 0.000 | 0.000 | A1 |
| Ots_P53-25 | One | 218 | G | T | 434 | 0 | 2 | 1.000 | 0.000 | 0.005 | A1 |
| Ots_P53-28 | One | 218 | G | C | 436 | 0 | 0 | 1.000 | 0.000 | 0.000 | A1 |
| Ots_P53-40 | One | 218 | T | C | 436 | 0 | 0 | 1.000 | 0.000 | 0.000 | A1 |
| Ots_unk9480-51-38 | One | 218 | C | T | 436 | 0 | 0 | 1.000 | 0.000 | 0.000 | A1 |

Table S8. Rainbow Trout/steelhead allele frequencies for the species informative markers. Estimates are derived using validation samples from Table S3. The Exp. Allele is the allele associated with the species as defined in the species-seq file.

| **Locus** | **Species** | ***N*** | **A1** | **A2** | **Count A1** | **Count A2** | **Count Miss.** | **A1 Freq.** | **A2 Freq.** | **Miss. Freq.** | **Exp. Allele** |
| --- | --- | --- | --- | --- | --- | --- | --- | --- | --- | --- | --- |
| Oki_101419-103-27 | Omy | 9755 | A | C | 19510 | 0 | 0 | 1.00000 | 0.00000 | 0.00000 | A1 |
| Oki_101419-103-44 | Omy | 9755 | T | C | 19510 | 0 | 0 | 1.00000 | 0.00000 | 0.00000 | A1 |
| Oki_105105-245-23 | Omy | 9755 | T | G | 19510 | 0 | 0 | 1.00000 | 0.00000 | 0.00000 | A1 |
| Oki_106172-60-38 | Omy | 9755 | T | C | 0 | 19500 | 10 | 0.00000 | 1.00000 | 0.00051 | A2 |
| Oki_106172-60-47 | Omy | 9755 | A | G | 626 | 18866 | 18 | 0.03212 | 0.96788 | 0.00092 | A2 |
| Oki_106172-60-47c | Omy | 9755 | A | C | 1238 | 0 | 18272 | 1.00000 | 0.00000 | 0.93655 | – |
| Oki_106172-60-53 | Omy | 9755 | T | C | 19510 | 0 | 0 | 1.00000 | 0.00000 | 0.00000 | A1 |
| Oki_111681-407-38 | Omy | 9755 | T | C | 7 | 19491 | 12 | 0.00036 | 0.99964 | 0.00062 | A2 |
| Oki_120255mod-105 | Omy | 9755 | C | T | 17978 | 0 | 1532 | 1.00000 | 0.00000 | 0.07852 | A1 |
| Oki_120255mod-113 | Omy | 9755 | A | G | 8 | 17776 | 1726 | 0.00045 | 0.99955 | 0.08847 | A2 |
| Oki_120255mod-115 | Omy | 9755 | G | T | 18024 | 0 | 1486 | 1.00000 | 0.00000 | 0.07617 | A1 |
| Oki_120255mod-119 | Omy | 9755 | G | A | 22 | 17756 | 1732 | 0.00124 | 0.99876 | 0.08877 | A2 |
| Oki_120255mod-120 | Omy | 9755 | C | T | 17999 | 1 | 1510 | 0.99994 | 0.00006 | 0.07740 | A1 |
| Oki_120255mod-133 | Omy | 9755 | T | C | 17958 | 0 | 1552 | 1.00000 | 0.00000 | 0.07955 | A1 |
| Oki_120255mod-135 | Omy | 9755 | A | C | 18000 | 0 | 1510 | 1.00000 | 0.00000 | 0.07740 | A1 |
| Oki_120255mod-137 | Omy | 9755 | A | T | 18014 | 0 | 1496 | 1.00000 | 0.00000 | 0.07668 | A1 |
| Oki_120255mod-141 | Omy | 9755 | A | G | 17990 | 0 | 1520 | 1.00000 | 0.00000 | 0.07791 | A1 |
| Oki_120255mod-99 | Omy | 9755 | A | C | 17992 | 0 | 1518 | 1.00000 | 0.00000 | 0.07781 | A1 |
| Oki_126619-265-31 | Omy | 9755 | A | C | 19510 | 0 | 0 | 1.00000 | 0.00000 | 0.00000 | A1 |
| Oki_126619-265-35 | Omy | 9755 | A | G | 19508 | 0 | 2 | 1.00000 | 0.00000 | 0.00010 | A1 |
| Oki_126619-265-50 | Omy | 9755 | G | T | 19510 | 0 | 0 | 1.00000 | 0.00000 | 0.00000 | A1 |
| Oki_aspAT-273-37 | Omy | 9755 | C | T | 19506 | 0 | 4 | 1.00000 | 0.00000 | 0.00021 | A1 |
| Oki_aspAT-273-45 | Omy | 9755 | T | A | 19480 | 0 | 30 | 1.00000 | 0.00000 | 0.00154 | A1 |
| Oki_RAD41030-31-36 | Omy | 9755 | C | T | 7844 | 0 | 11666 | 1.00000 | 0.00000 | 0.59795 | – |
| Oki_RAD41030-31-37 | Omy | 9755 | G | A | 9890 | 0 | 9620 | 1.00000 | 0.00000 | 0.49308 | – |
| Oki_RAD41030-31-41 | Omy | 9755 | G | C | 9703 | 7 | 9800 | 0.99928 | 0.00072 | 0.50231 | – |
| Oki_RAD41030-31-55 | Omy | 9755 | G | T | 3124 | 0 | 16386 | 1.00000 | 0.00000 | 0.83988 | – |
| Oki_RAD51585-47-25 | Omy | 9755 | C | G | 19380 | 2 | 128 | 0.99990 | 0.00010 | 0.00656 | A1 |
| Oki_RAD51585-47-28 | Omy | 9755 | G | A | 19382 | 0 | 128 | 1.00000 | 0.00000 | 0.00656 | A1 |
| Oki_RAD51585-47-31 | Omy | 9755 | A | C | 19396 | 0 | 114 | 1.00000 | 0.00000 | 0.00584 | A1 |
| Omy_myclarp404-111 | Omy | 9755 | G | T | 16 | 19460 | 34 | 0.00082 | 0.99918 | 0.00174 | A2 |
| Omy_RAD13034-67-21 | Omy | 9755 | T | C | 1 | 19489 | 20 | 0.00005 | 0.99995 | 0.00103 | A2 |
| Omy_RAD13034-67-35 | Omy | 9755 | A | T | 19492 | 14 | 4 | 0.99928 | 0.00072 | 0.00021 | A1 |
| Omy_RAD79314-58-25 | Omy | 9755 | C | T | 19492 | 18 | 0 | 0.99908 | 0.00092 | 0.00000 | A1 |
| Omy_RAD79314-58-27 | Omy | 9755 | C | T | 0 | 19496 | 14 | 0.00000 | 1.00000 | 0.00072 | A2 |
| Omy_RAD79314-58-31 | Omy | 9755 | G | A | 19510 | 0 | 0 | 1.00000 | 0.00000 | 0.00000 | A1 |
| Omy_RAD79314-58-66 | Omy | 9755 | C | A | 19510 | 0 | 0 | 1.00000 | 0.00000 | 0.00000 | A1 |
| One_1a.54542-52-44 | Omy | 9755 | C | T | 19510 | 0 | 0 | 1.00000 | 0.00000 | 0.00000 | A1 |
| One_1a.54542-52-47 | Omy | 9755 | G | C | 19510 | 0 | 0 | 1.00000 | 0.00000 | 0.00000 | A1 |
| One_2.70711-39-28 | Omy | 9755 | G | T | 19510 | 0 | 0 | 1.00000 | 0.00000 | 0.00000 | A1 |
| One_2.70711-39-30 | Omy | 9755 | T | C | 19510 | 0 | 0 | 1.00000 | 0.00000 | 0.00000 | A1 |
| One_2.70711-39-43 | Omy | 9755 | G | A | 19458 | 52 | 0 | 0.99733 | 0.00267 | 0.00000 | A1 |
| One_2.70711-39-57 | Omy | 9755 | G | A | 19510 | 0 | 0 | 1.00000 | 0.00000 | 0.00000 | A1 |
| One_2.70711-39-64 | Omy | 9755 | T | G | 19510 | 0 | 0 | 1.00000 | 0.00000 | 0.00000 | A1 |
| Ots_ARNT-29 | Omy | 9755 | G | A | 21 | 18715 | 774 | 0.00112 | 0.99888 | 0.03967 | A2 |
| Ots_ARNT-29c | Omy | 9755 | A | C | 19158 | 0 | 352 | 1.00000 | 0.00000 | 0.01804 | A1 |
| Ots_crRAD9615-69-19 | Omy | 9755 | T | A | 18840 | 26 | 644 | 0.99862 | 0.00138 | 0.03301 | A1 |
| Ots_crRAD9615-69-50 | Omy | 9755 | T | A | 3 | 19425 | 82 | 0.00015 | 0.99985 | 0.00420 | A2 |
| Ots_myo1a-384-36 | Omy | 9755 | C | T | 19510 | 0 | 0 | 1.00000 | 0.00000 | 0.00000 | A1 |
| Ots_P53-25 | Omy | 9755 | G | T | 19510 | 0 | 0 | 1.00000 | 0.00000 | 0.00000 | A1 |
| Ots_P53-28 | Omy | 9755 | G | C | 19510 | 0 | 0 | 1.00000 | 0.00000 | 0.00000 | A1 |
| Ots_P53-40 | Omy | 9755 | T | C | 19510 | 0 | 0 | 1.00000 | 0.00000 | 0.00000 | A1 |
| Ots_unk9480-51-38 | Omy | 9755 | C | T | 2 | 19496 | 12 | 0.00010 | 0.99990 | 0.00062 | A2 |

Table S9. Coastal Cutthroat Trout allele frequencies for the species informative markers. Estimates are derived using validation samples from Table S3. The Exp. Allele is the allele associated with the species as defined in the species-seq file.

| Locus | Species | N | A1 | A2 | Count A1 | Count A2 | Count Miss. | A1 Freq. | A2 Freq. | Miss. Freq. | Exp. Allele |
| --- | --- | --- | --- | --- | --- | --- | --- | --- | --- | --- | --- |
| Oki_101419-103-27 | Ocl1 | 21 | A | C | 42 | 0 | 0 | 1.0000 | 0.0000 | 0.0000 | A1 |
| Oki_101419-103-44 | Ocl1 | 21 | T | C | 42 | 0 | 0 | 1.0000 | 0.0000 | 0.0000 | A1 |
| Oki_105105-245-23 | Ocl1 | 21 | T | G | 42 | 0 | 0 | 1.0000 | 0.0000 | 0.0000 | A1 |
| Oki_106172-60-38 | Ocl1 | 21 | T | C | 0 | 42 | 0 | 0.0000 | 1.0000 | 0.0000 | A2 |
| Oki_106172-60-47 | Ocl1 | 21 | A | G | 0 | 42 | 0 | 0.0000 | 1.0000 | 0.0000 | A2 |
| Oki_106172-60-47c | Ocl1 | 21 | A | C | 0 | 0 | 42 | – | – | 1.0000 | – |
| Oki_106172-60-53 | Ocl1 | 21 | T | C | 42 | 0 | 0 | 1.0000 | 0.0000 | 0.0000 | A1 |
| Oki_111681-407-38 | Ocl1 | 21 | T | C | 0 | 42 | 0 | 0.0000 | 1.0000 | 0.0000 | A2 |
| Oki_120255mod-105 | Ocl1 | 21 | C | T | 28 | 0 | 14 | 1.0000 | 0.0000 | 0.3333 | A1 |
| Oki_120255mod-113 | Ocl1 | 21 | A | G | 0 | 28 | 14 | 0.0000 | 1.0000 | 0.3333 | A2 |
| Oki_120255mod-115 | Ocl1 | 21 | G | T | 28 | 0 | 14 | 1.0000 | 0.0000 | 0.3333 | A1 |
| Oki_120255mod-119 | Ocl1 | 21 | G | A | 28 | 0 | 14 | 1.0000 | 0.0000 | 0.3333 | A1 |
| Oki_120255mod-120 | Ocl1 | 21 | C | T | 28 | 0 | 14 | 1.0000 | 0.0000 | 0.3333 | A1 |
| Oki_120255mod-133 | Ocl1 | 21 | T | C | 28 | 0 | 14 | 1.0000 | 0.0000 | 0.3333 | A1 |
| Oki_120255mod-135 | Ocl1 | 21 | A | C | 28 | 0 | 14 | 1.0000 | 0.0000 | 0.3333 | A1 |
| Oki_120255mod-137 | Ocl1 | 21 | A | T | 28 | 0 | 14 | 1.0000 | 0.0000 | 0.3333 | A1 |
| Oki_120255mod-141 | Ocl1 | 21 | A | G | 28 | 0 | 14 | 1.0000 | 0.0000 | 0.3333 | A1 |
| Oki_120255mod-99 | Ocl1 | 21 | A | C | 28 | 0 | 14 | 1.0000 | 0.0000 | 0.3333 | A1 |
| Oki_126619-265-31 | Ocl1 | 21 | A | C | 42 | 0 | 0 | 1.0000 | 0.0000 | 0.0000 | A1 |
| Oki_126619-265-35 | Ocl1 | 21 | A | G | 42 | 0 | 0 | 1.0000 | 0.0000 | 0.0000 | A1 |
| Oki_126619-265-50 | Ocl1 | 21 | G | T | 42 | 0 | 0 | 1.0000 | 0.0000 | 0.0000 | A1 |
| Oki_RAD41030-31-36 | Ocl1 | 21 | C | T | 42 | 0 | 0 | 1.0000 | 0.0000 | 0.0000 | A1 |
| Oki_RAD41030-31-37 | Ocl1 | 21 | G | A | 42 | 0 | 0 | 1.0000 | 0.0000 | 0.0000 | A1 |
| Oki_RAD41030-31-41 | Ocl1 | 21 | G | C | 42 | 0 | 0 | 1.0000 | 0.0000 | 0.0000 | A1 |
| Oki_RAD41030-31-55 | Ocl1 | 21 | G | T | 42 | 0 | 0 | 1.0000 | 0.0000 | 0.0000 | A1 |
| Oki_RAD51585-47-25 | Ocl1 | 21 | C | G | 42 | 0 | 0 | 1.0000 | 0.0000 | 0.0000 | A1 |
| Oki_RAD51585-47-28 | Ocl1 | 21 | G | A | 42 | 0 | 0 | 1.0000 | 0.0000 | 0.0000 | A1 |
| Oki_RAD51585-47-31 | Ocl1 | 21 | A | C | 42 | 0 | 0 | 1.0000 | 0.0000 | 0.0000 | A1 |
| Oki_aspAT-273-37 | Ocl1 | 21 | C | T | 42 | 0 | 0 | 1.0000 | 0.0000 | 0.0000 | A1 |
| Oki_aspAT-273-45 | Ocl1 | 21 | T | A | 42 | 0 | 0 | 1.0000 | 0.0000 | 0.0000 | A1 |
| Omy_RAD13034-67-21 | Ocl1 | 21 | T | C | 0 | 42 | 0 | 0.0000 | 1.0000 | 0.0000 | A2 |
| Omy_RAD13034-67-35 | Ocl1 | 21 | A | T | 0 | 42 | 0 | 0.0000 | 1.0000 | 0.0000 | A2 |
| Omy_RAD79314-58-25 | Ocl1 | 21 | C | T | 42 | 0 | 0 | 1.0000 | 0.0000 | 0.0000 | A1 |
| Omy_RAD79314-58-27 | Ocl1 | 21 | C | T | 0 | 42 | 0 | 0.0000 | 1.0000 | 0.0000 | A2 |
| Omy_RAD79314-58-31 | Ocl1 | 21 | G | A | 42 | 0 | 0 | 1.0000 | 0.0000 | 0.0000 | A1 |
| Omy_RAD79314-58-66 | Ocl1 | 21 | C | A | 42 | 0 | 0 | 1.0000 | 0.0000 | 0.0000 | A1 |
| Omy_myclarp404-111 | Ocl1 | 21 | G | T | 40 | 0 | 2 | 1.0000 | 0.0000 | 0.0476 | A1 |
| One_1a.54542-52-44 | Ocl1 | 21 | C | T | 42 | 0 | 0 | 1.0000 | 0.0000 | 0.0000 | A1 |
| One_1a.54542-52-47 | Ocl1 | 21 | G | C | 42 | 0 | 0 | 1.0000 | 0.0000 | 0.0000 | A1 |
| One_2.70711-39-28 | Ocl1 | 21 | G | T | 42 | 0 | 0 | 1.0000 | 0.0000 | 0.0000 | A1 |
| One_2.70711-39-30 | Ocl1 | 21 | T | C | 42 | 0 | 0 | 1.0000 | 0.0000 | 0.0000 | A1 |
| One_2.70711-39-43 | Ocl1 | 21 | G | A | 42 | 0 | 0 | 1.0000 | 0.0000 | 0.0000 | A1 |
| One_2.70711-39-57 | Ocl1 | 21 | G | A | 42 | 0 | 0 | 1.0000 | 0.0000 | 0.0000 | A1 |
| One_2.70711-39-64 | Ocl1 | 21 | T | G | 42 | 0 | 0 | 1.0000 | 0.0000 | 0.0000 | A1 |
| Ots_ARNT-29 | Ocl1 | 21 | G | A | 0 | 42 | 0 | 0.0000 | 1.0000 | 0.0000 | A2 |
| Ots_ARNT-29c | Ocl1 | 21 | A | C | 42 | 0 | 0 | 1.0000 | 0.0000 | 0.0000 | A1 |
| Ots_P53-25 | Ocl1 | 21 | G | T | 42 | 0 | 0 | 1.0000 | 0.0000 | 0.0000 | A1 |
| Ots_P53-28 | Ocl1 | 21 | G | C | 42 | 0 | 0 | 1.0000 | 0.0000 | 0.0000 | A1 |
| Ots_P53-40 | Ocl1 | 21 | T | C | 42 | 0 | 0 | 1.0000 | 0.0000 | 0.0000 | A1 |
| Ots_crRAD9615-69-19 | Ocl1 | 21 | T | A | 42 | 0 | 0 | 1.0000 | 0.0000 | 0.0000 | A1 |
| Ots_crRAD9615-69-50 | Ocl1 | 21 | T | A | 0 | 42 | 0 | 0.0000 | 1.0000 | 0.0000 | A2 |
| Ots_myo1a-384-36 | Ocl1 | 21 | C | T | 40 | 0 | 2 | 1.0000 | 0.0000 | 0.0476 | A1 |
| Ots_unk9480-51-38 | Ocl1 | 21 | C | T | 0 | 42 | 0 | 0.0000 | 1.0000 | 0.0000 | A2 |

Table S10. Bonneville and Yellowstone Cutthroat Trout allele frequencies for the species informative markers. Estimates are derived using validation samples from Table S3. The Exp. Allele is the allele associated with the species as defined in the species-seq file.

| **Locus** | **Species** | ***N*** | **A1** | **A2** | **Count A1** | **Count A2** | **Count Miss.** | **A1 Freq.** | **A2 Freq.** | **Miss. Freq.** | **Exp. Allele** |
| --- | --- | --- | --- | --- | --- | --- | --- | --- | --- | --- | --- |
| Oki_101419-103-27 | Ocl2 | 16 | A | C | 32 | 0 | 0 | 1.0000 | 0.0000 | 0.0000 | A1 |
| Oki_101419-103-44 | Ocl2 | 16 | T | C | 32 | 0 | 0 | 1.0000 | 0.0000 | 0.0000 | A1 |
| Oki_105105-245-23 | Ocl2 | 16 | T | G | 32 | 0 | 0 | 1.0000 | 0.0000 | 0.0000 | A1 |
| Oki_106172-60-38 | Ocl2 | 16 | T | C | 0 | 32 | 0 | 0.0000 | 1.0000 | 0.0000 | A2 |
| Oki_106172-60-47 | Ocl2 | 16 | A | G | 32 | 0 | 0 | 1.0000 | 0.0000 | 0.0000 | A1 |
| Oki_106172-60-47c | Ocl2 | 16 | A | C | 32 | 0 | 0 | 1.0000 | 0.0000 | 0.0000 | A1 |
| Oki_106172-60-53 | Ocl2 | 16 | T | C | 32 | 0 | 0 | 1.0000 | 0.0000 | 0.0000 | A1 |
| Oki_111681-407-38 | Ocl2 | 16 | T | C | 0 | 32 | 0 | 0.0000 | 1.0000 | 0.0000 | A2 |
| Oki_120255mod-105 | Ocl2 | 16 | C | T | 32 | 0 | 0 | 1.0000 | 0.0000 | 0.0000 | A1 |
| Oki_120255mod-113 | Ocl2 | 16 | A | G | 0 | 32 | 0 | 0.0000 | 1.0000 | 0.0000 | A2 |
| Oki_120255mod-115 | Ocl2 | 16 | G | T | 32 | 0 | 0 | 1.0000 | 0.0000 | 0.0000 | A1 |
| Oki_120255mod-119 | Ocl2 | 16 | G | A | 32 | 0 | 0 | 1.0000 | 0.0000 | 0.0000 | A1 |
| Oki_120255mod-120 | Ocl2 | 16 | C | T | 32 | 0 | 0 | 1.0000 | 0.0000 | 0.0000 | A1 |
| Oki_120255mod-133 | Ocl2 | 16 | T | C | 32 | 0 | 0 | 1.0000 | 0.0000 | 0.0000 | A1 |
| Oki_120255mod-135 | Ocl2 | 16 | A | C | 32 | 0 | 0 | 1.0000 | 0.0000 | 0.0000 | A1 |
| Oki_120255mod-137 | Ocl2 | 16 | A | T | 32 | 0 | 0 | 1.0000 | 0.0000 | 0.0000 | A1 |
| Oki_120255mod-141 | Ocl2 | 16 | A | G | 32 | 0 | 0 | 1.0000 | 0.0000 | 0.0000 | A1 |
| Oki_120255mod-99 | Ocl2 | 16 | A | C | 32 | 0 | 0 | 1.0000 | 0.0000 | 0.0000 | A1 |
| Oki_126619-265-31 | Ocl2 | 16 | A | C | 32 | 0 | 0 | 1.0000 | 0.0000 | 0.0000 | A1 |
| Oki_126619-265-35 | Ocl2 | 16 | A | G | 32 | 0 | 0 | 1.0000 | 0.0000 | 0.0000 | A1 |
| Oki_126619-265-50 | Ocl2 | 16 | G | T | 32 | 0 | 0 | 1.0000 | 0.0000 | 0.0000 | A1 |
| Oki_aspAT-273-37 | Ocl2 | 16 | C | T | 32 | 0 | 0 | 1.0000 | 0.0000 | 0.0000 | A1 |
| Oki_aspAT-273-45 | Ocl2 | 16 | T | A | 32 | 0 | 0 | 1.0000 | 0.0000 | 0.0000 | A1 |
| Oki_RAD41030-31-36 | Ocl2 | 16 | C | T | 0 | 32 | 0 | 0.0000 | 1.0000 | 0.0000 | A2 |
| Oki_RAD41030-31-37 | Ocl2 | 16 | G | A | 32 | 0 | 0 | 1.0000 | 0.0000 | 0.0000 | A1 |
| Oki_RAD41030-31-41 | Ocl2 | 16 | G | C | 32 | 0 | 0 | 1.0000 | 0.0000 | 0.0000 | A1 |
| Oki_RAD41030-31-55 | Ocl2 | 16 | G | T | 32 | 0 | 0 | 1.0000 | 0.0000 | 0.0000 | A1 |
| Oki_RAD51585-47-25 | Ocl2 | 16 | C | G | 32 | 0 | 0 | 1.0000 | 0.0000 | 0.0000 | A1 |
| Oki_RAD51585-47-28 | Ocl2 | 16 | G | A | 0 | 0 | 32 | – | – | 1.0000 | – |
| Oki_RAD51585-47-31 | Ocl2 | 16 | A | C | 0 | 32 | 0 | 0.0000 | 1.0000 | 0.0000 | A2 |
| Omy_myclarp404-111 | Ocl2 | 16 | G | T | 32 | 0 | 0 | 1.0000 | 0.0000 | 0.0000 | A1 |
| Omy_RAD13034-67-21 | Ocl2 | 16 | T | C | 0 | 32 | 0 | 0.0000 | 1.0000 | 0.0000 | A2 |
| Omy_RAD13034-67-35 | Ocl2 | 16 | A | T | 0 | 32 | 0 | 0.0000 | 1.0000 | 0.0000 | A2 |
| Omy_RAD79314-58-25 | Ocl2 | 16 | C | T | 32 | 0 | 0 | 1.0000 | 0.0000 | 0.0000 | A1 |
| Omy_RAD79314-58-27 | Ocl2 | 16 | C | T | 0 | 32 | 0 | 0.0000 | 1.0000 | 0.0000 | A2 |
| Omy_RAD79314-58-31 | Ocl2 | 16 | G | A | 32 | 0 | 0 | 1.0000 | 0.0000 | 0.0000 | A1 |
| Omy_RAD79314-58-66 | Ocl2 | 16 | C | A | 32 | 0 | 0 | 1.0000 | 0.0000 | 0.0000 | A1 |
| One_1a.54542-52-44 | Ocl2 | 16 | C | T | 32 | 0 | 0 | 1.0000 | 0.0000 | 0.0000 | A1 |
| One_1a.54542-52-47 | Ocl2 | 16 | G | C | 32 | 0 | 0 | 1.0000 | 0.0000 | 0.0000 | A1 |
| One_2.70711-39-28 | Ocl2 | 16 | G | T | 32 | 0 | 0 | 1.0000 | 0.0000 | 0.0000 | A1 |
| One_2.70711-39-30 | Ocl2 | 16 | T | C | 32 | 0 | 0 | 1.0000 | 0.0000 | 0.0000 | A1 |
| One_2.70711-39-43 | Ocl2 | 16 | G | A | 32 | 0 | 0 | 1.0000 | 0.0000 | 0.0000 | A1 |
| One_2.70711-39-57 | Ocl2 | 16 | G | A | 32 | 0 | 0 | 1.0000 | 0.0000 | 0.0000 | A1 |
| One_2.70711-39-64 | Ocl2 | 16 | T | G | 32 | 0 | 0 | 1.0000 | 0.0000 | 0.0000 | A1 |
| Ots_ARNT-29 | Ocl2 | 16 | G | A | 0 | 32 | 0 | 0.0000 | 1.0000 | 0.0000 | A2 |
| Ots_ARNT-29c | Ocl2 | 16 | A | C | 32 | 0 | 0 | 1.0000 | 0.0000 | 0.0000 | A1 |
| Ots_crRAD9615-69-19 | Ocl2 | 16 | T | A | 32 | 0 | 0 | 1.0000 | 0.0000 | 0.0000 | A1 |
| Ots_crRAD9615-69-50 | Ocl2 | 16 | T | A | 0 | 32 | 0 | 0.0000 | 1.0000 | 0.0000 | A2 |
| Ots_myo1a-384-36 | Ocl2 | 16 | C | T | 0 | 32 | 0 | 0.0000 | 1.0000 | 0.0000 | A2 |
| Ots_P53-25 | Ocl2 | 16 | G | T | 32 | 0 | 0 | 1.0000 | 0.0000 | 0.0000 | A1 |
| Ots_P53-28 | Ocl2 | 16 | G | C | 32 | 0 | 0 | 1.0000 | 0.0000 | 0.0000 | A1 |
| Ots_P53-40 | Ocl2 | 16 | T | C | 32 | 0 | 0 | 1.0000 | 0.0000 | 0.0000 | A1 |
| Ots_unk9480-51-38 | Ocl2 | 16 | C | T | 0 | 32 | 0 | 0.0000 | 1.0000 | 0.0000 | A2 |

Table S11 Bull Trout allele frequencies for the species informative markers. Estimates are derived using validation samples from Table S3. The Exp. Allele is the allele associated with the species as defined in the species-seq file.

| **Locus** | **Species** | ***N*** | **A1** | **A2** | **Count A1** | **Count A2** | **Count Miss.** | **A1 Freq.** | **A2 Freq.** | **Miss. Freq.** | **Exp. Allele** |
| --- | --- | --- | --- | --- | --- | --- | --- | --- | --- | --- | --- |
| Oki_101419-103-27 | Sco | 16 | A | C | 0 | 0 | 32 | – | – | 1.0000 | – |
| Oki_101419-103-44 | Sco | 16 | T | C | 0 | 0 | 32 | – | – | 1.0000 | – |
| Oki_105105-245-23 | Sco | 16 | T | G | 32 | 0 | 0 | 1.0000 | 0.0000 | 0.0000 | A1 |
| Oki_106172-60-38 | Sco | 16 | T | C | 32 | 0 | 0 | 1.0000 | 0.0000 | 0.0000 | A1 |
| Oki_106172-60-47 | Sco | 16 | A | G | 0 | 0 | 32 | – | – | 1.0000 | – |
| Oki_106172-60-47c | Sco | 16 | A | C | 0 | 32 | 0 | 0.0000 | 1.0000 | 0.0000 | A2 |
| Oki_106172-60-53 | Sco | 16 | T | C | 32 | 0 | 0 | 1.0000 | 0.0000 | 0.0000 | A1 |
| Oki_111681-407-38 | Sco | 16 | T | C | 0 | 32 | 0 | 0.0000 | 1.0000 | 0.0000 | A2 |
| Oki_120255mod-105 | Sco | 16 | C | T | 30 | 0 | 2 | 1.0000 | 0.0000 | 0.0625 | A1 |
| Oki_120255mod-113 | Sco | 16 | A | G | 0 | 30 | 2 | 0.0000 | 1.0000 | 0.0625 | A2 |
| Oki_120255mod-115 | Sco | 16 | G | T | 30 | 0 | 2 | 1.0000 | 0.0000 | 0.0625 | A1 |
| Oki_120255mod-119 | Sco | 16 | G | A | 30 | 0 | 2 | 1.0000 | 0.0000 | 0.0625 | A1 |
| Oki_120255mod-120 | Sco | 16 | C | T | 30 | 0 | 2 | 1.0000 | 0.0000 | 0.0625 | A1 |
| Oki_120255mod-133 | Sco | 16 | T | C | 30 | 0 | 2 | 1.0000 | 0.0000 | 0.0625 | A1 |
| Oki_120255mod-135 | Sco | 16 | A | C | 30 | 0 | 2 | 1.0000 | 0.0000 | 0.0625 | A1 |
| Oki_120255mod-137 | Sco | 16 | A | T | 30 | 0 | 2 | 1.0000 | 0.0000 | 0.0625 | A1 |
| Oki_120255mod-141 | Sco | 16 | A | G | 30 | 0 | 2 | 1.0000 | 0.0000 | 0.0625 | A1 |
| Oki_120255mod-99 | Sco | 16 | A | C | 30 | 0 | 2 | 1.0000 | 0.0000 | 0.0625 | A1 |
| Oki_126619-265-31 | Sco | 16 | A | C | 30 | 0 | 2 | 1.0000 | 0.0000 | 0.0625 | A1 |
| Oki_126619-265-35 | Sco | 16 | A | G | 30 | 0 | 2 | 1.0000 | 0.0000 | 0.0625 | A1 |
| Oki_126619-265-50 | Sco | 16 | G | T | 30 | 0 | 2 | 1.0000 | 0.0000 | 0.0625 | A1 |
| Oki_RAD41030-31-36 | Sco | 16 | C | T | 32 | 0 | 0 | 1.0000 | 0.0000 | 0.0000 | A1 |
| Oki_RAD41030-31-37 | Sco | 16 | G | A | 0 | 32 | 0 | 0.0000 | 1.0000 | 0.0000 | A2 |
| Oki_RAD41030-31-41 | Sco | 16 | G | C | 32 | 0 | 0 | 1.0000 | 0.0000 | 0.0000 | A1 |
| Oki_RAD41030-31-55 | Sco | 16 | G | T | 32 | 0 | 0 | 1.0000 | 0.0000 | 0.0000 | A1 |
| Oki_RAD51585-47-25 | Sco | 16 | C | G | 0 | 0 | 32 | – | – | 1.0000 | A2 |
| Oki_RAD51585-47-28 | Sco | 16 | G | A | 0 | 0 | 32 | – | – | 1.0000 | – |
| Oki_RAD51585-47-31 | Sco | 16 | A | C | 0 | 0 | 32 | – | – | 1.0000 | A1 |
| Oki_aspAT-273-37 | Sco | 16 | C | T | 32 | 0 | 0 | 1.0000 | 0.0000 | 0.0000 | A1 |
| Oki_aspAT-273-45 | Sco | 16 | T | A | 0 | 32 | 0 | 0.0000 | 1.0000 | 0.0000 | A2 |
| Omy_RAD13034-67-21 | Sco | 16 | T | C | 0 | 0 | 32 | – | – | 1.0000 | – |
| Omy_RAD13034-67-35 | Sco | 16 | A | T | 0 | 0 | 32 | – | – | 1.0000 | – |
| Omy_RAD79314-58-25 | Sco | 16 | C | T | 32 | 0 | 0 | 1.0000 | 0.0000 | 0.0000 | A1 |
| Omy_RAD79314-58-27 | Sco | 16 | C | T | 32 | 0 | 0 | 1.0000 | 0.0000 | 0.0000 | A1 |
| Omy_RAD79314-58-31 | Sco | 16 | G | A | 32 | 0 | 0 | 1.0000 | 0.0000 | 0.0000 | A1 |
| Omy_RAD79314-58-66 | Sco | 16 | C | A | 32 | 0 | 0 | 1.0000 | 0.0000 | 0.0000 | A1 |
| Omy_myclarp404-111 | Sco | 16 | G | T | 32 | 0 | 0 | 1.0000 | 0.0000 | 0.0000 | A1 |
| One_1a.54542-52-44 | Sco | 16 | C | T | 0 | 28 | 4 | 0.0000 | 1.0000 | 0.1250 | A2 |
| One_1a.54542-52-47 | Sco | 16 | G | C | 28 | 0 | 4 | 1.0000 | 0.0000 | 0.1250 | A1 |
| One_2.70711-39-28 | Sco | 16 | G | T | 32 | 0 | 0 | 1.0000 | 0.0000 | 0.0000 | A1 |
| One_2.70711-39-30 | Sco | 16 | T | C | 0 | 32 | 0 | 0.0000 | 1.0000 | 0.0000 | A2 |
| One_2.70711-39-43 | Sco | 16 | G | A | 32 | 0 | 0 | 1.0000 | 0.0000 | 0.0000 | A1 |
| One_2.70711-39-57 | Sco | 16 | G | A | 32 | 0 | 0 | 1.0000 | 0.0000 | 0.0000 | A1 |
| One_2.70711-39-64 | Sco | 16 | T | G | 32 | 0 | 0 | 1.0000 | 0.0000 | 0.0000 | A1 |
| Ots_ARNT-29 | Sco | 16 | G | A | 30 | 0 | 2 | 1.0000 | 0.0000 | 0.0625 | A1 |
| Ots_ARNT-29c | Sco | 16 | A | C | 0 | 0 | 32 | – | – | 1.0000 | – |
| Ots_P53-25 | Sco | 16 | G | T | 30 | 0 | 2 | 1.0000 | 0.0000 | 0.0625 | A1 |
| Ots_P53-28 | Sco | 16 | G | C | 0 | 30 | 2 | 0.0000 | 1.0000 | 0.0625 | A2 |
| Ots_P53-40 | Sco | 16 | T | C | 0 | 30 | 2 | 0.0000 | 1.0000 | 0.0625 | A2 |
| Ots_crRAD9615-69-19 | Sco | 16 | T | A | 30 | 0 | 2 | 1.0000 | 0.0000 | 0.0625 | A1 |
| Ots_crRAD9615-69-50 | Sco | 16 | T | A | 0 | 30 | 2 | 0.0000 | 1.0000 | 0.0625 | A2 |
| Ots_myo1a-384-36 | Sco | 16 | C | T | 0 | 0 | 32 | – | – | 1.0000 | – |
| Ots_unk9480-51-38 | Sco | 16 | C | T | 0 | 0 | 32 | – | – | 1.0000 | – |

Table S12. Brook Trout allele frequencies for the species informative markers. Estimates are derived using validation samples from Table S3.

| **Locus** | **Species** | ***N*** | **A1** | **A2** | **Count A1** | **Count A2** | **Count Miss.** | **A1 Freq.** | **A2 Freq.** | **Miss. Freq.** | **Exp. Allele** |
| --- | --- | --- | --- | --- | --- | --- | --- | --- | --- | --- | --- |
| Oki_101419-103-27 | Sfo | 16 | A | C | 0 | 32 | 0 | 0.0000 | 1.0000 | 0.0000 | A2 |
| Oki_101419-103-44 | Sfo | 16 | T | C | 0 | 32 | 0 | 0.0000 | 1.0000 | 0.0000 | A2 |
| Oki_105105-245-23 | Sfo | 16 | T | G | 32 | 0 | 0 | 1.0000 | 0.0000 | 0.0000 | A1 |
| Oki_106172-60-38 | Sfo | 16 | T | C | 32 | 0 | 0 | 1.0000 | 0.0000 | 0.0000 | A1 |
| Oki_106172-60-47 | Sfo | 16 | A | G | 0 | 0 | 32 | – | – | 1.0000 | – |
| Oki_106172-60-47c | Sfo | 16 | A | C | 0 | 32 | 0 | 0.0000 | 1.0000 | 0.0000 | A2 |
| Oki_106172-60-53 | Sfo | 16 | T | C | 32 | 0 | 0 | 1.0000 | 0.0000 | 0.0000 | A1 |
| Oki_111681-407-38 | Sfo | 16 | T | C | 0 | 32 | 0 | 0.0000 | 1.0000 | 0.0000 | A2 |
| Oki_120255mod-105 | Sfo | 16 | C | T | 32 | 0 | 0 | 1.0000 | 0.0000 | 0.0000 | A1 |
| Oki_120255mod-113 | Sfo | 16 | A | G | 0 | 32 | 0 | 0.0000 | 1.0000 | 0.0000 | A2 |
| Oki_120255mod-115 | Sfo | 16 | G | T | 32 | 0 | 0 | 1.0000 | 0.0000 | 0.0000 | A1 |
| Oki_120255mod-119 | Sfo | 16 | G | A | 32 | 0 | 0 | 1.0000 | 0.0000 | 0.0000 | A1 |
| Oki_120255mod-120 | Sfo | 16 | C | T | 0 | 32 | 0 | 0.0000 | 1.0000 | 0.0000 | A2 |
| Oki_120255mod-133 | Sfo | 16 | T | C | 32 | 0 | 0 | 1.0000 | 0.0000 | 0.0000 | A1 |
| Oki_120255mod-135 | Sfo | 16 | A | C | 32 | 0 | 0 | 1.0000 | 0.0000 | 0.0000 | A1 |
| Oki_120255mod-137 | Sfo | 16 | A | T | 32 | 0 | 0 | 1.0000 | 0.0000 | 0.0000 | A1 |
| Oki_120255mod-141 | Sfo | 16 | A | G | 31 | 1 | 0 | 0.9688 | 0.0313 | 0.0000 | A1 |
| Oki_120255mod-99 | Sfo | 16 | A | C | 32 | 0 | 0 | 1.0000 | 0.0000 | 0.0000 | A1 |
| Oki_126619-265-31 | Sfo | 16 | A | C | 0 | 22 | 10 | 0.0000 | 1.0000 | 0.3125 | A2 |
| Oki_126619-265-35 | Sfo | 16 | A | G | 20 | 0 | 12 | 1.0000 | 0.0000 | 0.3750 | A1 |
| Oki_126619-265-50 | Sfo | 16 | G | T | 20 | 0 | 12 | 1.0000 | 0.0000 | 0.3750 | A1 |
| Oki_RAD41030-31-36 | Sfo | 16 | C | T | 32 | 0 | 0 | 1.0000 | 0.0000 | 0.0000 | A1 |
| Oki_RAD41030-31-37 | Sfo | 16 | G | A | 0 | 32 | 0 | 0.0000 | 1.0000 | 0.0000 | A2 |
| Oki_RAD41030-31-41 | Sfo | 16 | G | C | 32 | 0 | 0 | 1.0000 | 0.0000 | 0.0000 | A1 |
| Oki_RAD41030-31-55 | Sfo | 16 | G | T | 32 | 0 | 0 | 1.0000 | 0.0000 | 0.0000 | A1 |
| Oki_RAD51585-47-25 | Sfo | 16 | C | G | 0 | 8 | 24 | 0.0000 | 1.0000 | 0.7500 | A2 |
| Oki_RAD51585-47-28 | Sfo | 16 | G | A | 0 | 0 | 32 | – | – | 1.0000 | – |
| Oki_RAD51585-47-31 | Sfo | 16 | A | C | 2 | 0 | 30 | 1.0000 | 0.0000 | 0.9375 | A1 |
| Oki_aspAT-273-37 | Sfo | 16 | C | T | 32 | 0 | 0 | 1.0000 | 0.0000 | 0.0000 | A1 |
| Oki_aspAT-273-45 | Sfo | 16 | T | A | 0 | 32 | 0 | 0.0000 | 1.0000 | 0.0000 | A2 |
| Omy_RAD13034-67-21 | Sfo | 16 | T | C | 0 | 0 | 32 | – | – | 1.0000 | – |
| Omy_RAD13034-67-35 | Sfo | 16 | A | T | 0 | 0 | 32 | – | – | 1.0000 | – |
| Omy_RAD79314-58-25 | Sfo | 16 | C | T | 32 | 0 | 0 | 1.0000 | 0.0000 | 0.0000 | A1 |
| Omy_RAD79314-58-27 | Sfo | 16 | C | T | 32 | 0 | 0 | 1.0000 | 0.0000 | 0.0000 | A1 |
| Omy_RAD79314-58-31 | Sfo | 16 | G | A | 32 | 0 | 0 | 1.0000 | 0.0000 | 0.0000 | A1 |
| Omy_RAD79314-58-66 | Sfo | 16 | C | A | 32 | 0 | 0 | 1.0000 | 0.0000 | 0.0000 | A1 |
| Omy_myclarp404-111 | Sfo | 16 | G | T | 32 | 0 | 0 | 1.0000 | 0.0000 | 0.0000 | A1 |
| One_1a.54542-52-44 | Sfo | 16 | C | T | 0 | 32 | 0 | 0.0000 | 1.0000 | 0.0000 | A2 |
| One_1a.54542-52-47 | Sfo | 16 | G | C | 32 | 0 | 0 | 1.0000 | 0.0000 | 0.0000 | A1 |
| One_2.70711-39-28 | Sfo | 16 | G | T | 16 | 0 | 16 | 1.0000 | 0.0000 | 0.5000 | A1 |
| One_2.70711-39-30 | Sfo | 16 | T | C | 0 | 16 | 16 | 0.0000 | 1.0000 | 0.5000 | A2 |
| One_2.70711-39-43 | Sfo | 16 | G | A | 26 | 0 | 6 | 1.0000 | 0.0000 | 0.1875 | A1 |
| One_2.70711-39-57 | Sfo | 16 | G | A | 26 | 0 | 6 | 1.0000 | 0.0000 | 0.1875 | A1 |
| One_2.70711-39-64 | Sfo | 16 | T | G | 26 | 0 | 6 | 1.0000 | 0.0000 | 0.1875 | A1 |
| Ots_ARNT-29 | Sfo | 16 | G | A | 32 | 0 | 0 | 1.0000 | 0.0000 | 0.0000 | A1 |
| Ots_ARNT-29c | Sfo | 16 | A | C | 0 | 0 | 32 | – | – | 1.0000 | – |
| Ots_P53-25 | Sfo | 16 | G | T | 32 | 0 | 0 | 1.0000 | 0.0000 | 0.0000 | A1 |
| Ots_P53-28 | Sfo | 16 | G | C | 32 | 0 | 0 | 1.0000 | 0.0000 | 0.0000 | A1 |
| Ots_P53-40 | Sfo | 16 | T | C | 0 | 32 | 0 | 0.0000 | 1.0000 | 0.0000 | A2 |
| Ots_crRAD9615-69-19 | Sfo | 16 | T | A | 32 | 0 | 0 | 1.0000 | 0.0000 | 0.0000 | A1 |
| Ots_crRAD9615-69-50 | Sfo | 16 | T | A | 0 | 32 | 0 | 0.0000 | 1.0000 | 0.0000 | A2 |
| Ots_myo1a-384-36 | Sfo | 16 | C | T | 0 | 0 | 32 | – | – | 1.0000 | – |
| Ots_unk9480-51-38 | Sfo | 16 | C | T | 0 | 0 | 32 | – | – | 1.0000 | – |

Table S13. Brown Trout allele frequencies for the species informative markers. Estimates are derived using validation samples from Table S3.

| Locus | Species | *N* | A1 | A2 | Count A1 | Count A2 | Count Miss. | A1 Freq. | A2 Freq. | Miss. Freq. | Exp. Allele |
| --- | --- | --- | --- | --- | --- | --- | --- | --- | --- | --- | --- |
| Oki_101419-103-27 | Str | 15 | A | C | 0 | 30 | 0 | 0.00000 | 1.00000 | 0.00000 | A2 |
| Oki_101419-103-44 | Str | 15 | T | C | 0 | 30 | 0 | 0.00000 | 1.00000 | 0.00000 | A2 |
| Oki_105105-245-23 | Str | 15 | T | G | 30 | 0 | 0 | 1.00000 | 0.00000 | 0.00000 | A1 |
| Oki_106172-60-38 | Str | 15 | T | C | 30 | 0 | 0 | 1.00000 | 0.00000 | 0.00000 | A1 |
| Oki_106172-60-47 | Str | 15 | A | G | 0 | 0 | 30 | – | – | 1.00000 | – |
| Oki_106172-60-47c | Str | 15 | A | C | 0 | 30 | 0 | 0.00000 | 1.00000 | 0.00000 | A2 |
| Oki_106172-60-53 | Str | 15 | T | C | 30 | 0 | 0 | 1.00000 | 0.00000 | 0.00000 | A1 |
| Oki_111681-407-38 | Str | 15 | T | C | 0 | 30 | 0 | 0.00000 | 1.00000 | 0.00000 | A2 |
| Oki_120255mod-105 | Str | 15 | C | T | 30 | 0 | 0 | 1.00000 | 0.00000 | 0.00000 | A1 |
| Oki_120255mod-113 | Str | 15 | A | G | 0 | 30 | 0 | 0.00000 | 1.00000 | 0.00000 | A2 |
| Oki_120255mod-115 | Str | 15 | G | T | 30 | 0 | 0 | 1.00000 | 0.00000 | 0.00000 | A1 |
| Oki_120255mod-119 | Str | 15 | G | A | 30 | 0 | 0 | 1.00000 | 0.00000 | 0.00000 | A1 |
| Oki_120255mod-120 | Str | 15 | C | T | 30 | 0 | 0 | 1.00000 | 0.00000 | 0.00000 | A1 |
| Oki_120255mod-133 | Str | 15 | T | C | 30 | 0 | 0 | 1.00000 | 0.00000 | 0.00000 | A1 |
| Oki_120255mod-135 | Str | 15 | A | C | 30 | 0 | 0 | 1.00000 | 0.00000 | 0.00000 | A1 |
| Oki_120255mod-137 | Str | 15 | A | T | 30 | 0 | 0 | 1.00000 | 0.00000 | 0.00000 | A1 |
| Oki_120255mod-141 | Str | 15 | A | G | 30 | 0 | 0 | 1.00000 | 0.00000 | 0.00000 | A1 |
| Oki_120255mod-99 | Str | 15 | A | C | 30 | 0 | 0 | 1.00000 | 0.00000 | 0.00000 | A1 |
| Oki_126619-265-31 | Str | 15 | A | C | 0 | 0 | 30 | – | – | 1.00000 | – |
| Oki_126619-265-35 | Str | 15 | A | G | 0 | 0 | 30 | – | – | 1.00000 | – |
| Oki_126619-265-50 | Str | 15 | G | T | 0 | 0 | 30 | – | – | 1.00000 | – |
| Oki_RAD41030-31-36 | Str | 15 | C | T | 30 | 0 | 0 | 1.00000 | 0.00000 | 0.00000 | A1 |
| Oki_RAD41030-31-37 | Str | 15 | G | A | 30 | 0 | 0 | 1.00000 | 0.00000 | 0.00000 | A1 |
| Oki_RAD41030-31-41 | Str | 15 | G | C | 30 | 0 | 0 | 1.00000 | 0.00000 | 0.00000 | A1 |
| Oki_RAD41030-31-55 | Str | 15 | G | T | 30 | 0 | 0 | 1.00000 | 0.00000 | 0.00000 | A1 |
| Oki_RAD51585-47-25 | Str | 15 | C | G | 0 | 0 | 30 | – | – | 1.00000 | A2 |
| Oki_RAD51585-47-28 | Str | 15 | G | A | 0 | 0 | 30 | – | – | 1.00000 | A1 |
| Oki_RAD51585-47-31 | Str | 15 | A | C | 25 | 1 | 4 | 0.96154 | 0.03846 | 0.13333 | A1 |
| Oki_aspAT-273-37 | Str | 15 | C | T | 30 | 0 | 0 | 1.00000 | 0.00000 | 0.00000 | A1 |
| Oki_aspAT-273-45 | Str | 15 | T | A | 0 | 0 | 30 | – | – | 1.00000 | – |
| Omy_RAD13034-67-21 | Str | 15 | T | C | 0 | 0 | 30 | – | – | 1.00000 | – |
| Omy_RAD13034-67-35 | Str | 15 | A | T | 0 | 0 | 30 | – | – | 1.00000 | – |
| Omy_RAD79314-58-25 | Str | 15 | C | T | 0 | 30 | 0 | 0.00000 | 1.00000 | 0.00000 | A2 |
| Omy_RAD79314-58-27 | Str | 15 | C | T | 30 | 0 | 0 | 1.00000 | 0.00000 | 0.00000 | A1 |
| Omy_RAD79314-58-31 | Str | 15 | G | A | 30 | 0 | 0 | 1.00000 | 0.00000 | 0.00000 | A1 |
| Omy_RAD79314-58-66 | Str | 15 | C | A | 0 | 30 | 0 | 0.00000 | 1.00000 | 0.00000 | A2 |
| Omy_myclarp404-111 | Str | 15 | G | T | 30 | 0 | 0 | 1.00000 | 0.00000 | 0.00000 | A1 |
| One_1a.54542-52-44 | Str | 15 | C | T | 0 | 28 | 2 | 0.00000 | 1.00000 | 0.06667 | A2 |
| One_1a.54542-52-47 | Str | 15 | G | C | 28 | 0 | 2 | 1.00000 | 0.00000 | 0.06667 | A1 |
| One_2.70711-39-28 | Str | 15 | G | T | 8 | 0 | 22 | 1.00000 | 0.00000 | 0.73333 | A1 |
| One_2.70711-39-30 | Str | 15 | T | C | 1 | 7 | 22 | 0.12500 | 0.87500 | 0.73333 | – |
| One_2.70711-39-43 | Str | 15 | G | A | 1 | 7 | 22 | 0.12500 | 0.87500 | 0.73333 | – |
| One_2.70711-39-57 | Str | 15 | G | A | 8 | 0 | 22 | 1.00000 | 0.00000 | 0.73333 | A1 |
| One_2.70711-39-64 | Str | 15 | T | G | 8 | 0 | 22 | 1.00000 | 0.00000 | 0.73333 | A1 |
| Ots_ARNT-29 | Str | 15 | G | A | 0 | 0 | 30 | – | – | 1.00000 | – |
| Ots_ARNT-29c | Str | 15 | A | C | 0 | 0 | 30 | – | – | 1.00000 | – |
| Ots_P53-25 | Str | 15 | G | T | 28 | 0 | 2 | 1.00000 | 0.00000 | 0.06667 | A1 |
| Ots_P53-28 | Str | 15 | G | C | 24 | 0 | 6 | 1.00000 | 0.00000 | 0.20000 | A1 |
| Ots_P53-40 | Str | 15 | T | C | 0 | 24 | 6 | 0.00000 | 1.00000 | 0.20000 | A2 |
| Ots_crRAD9615-69-19 | Str | 15 | T | A | 30 | 0 | 0 | 1.00000 | 0.00000 | 0.00000 | A1 |
| Ots_crRAD9615-69-50 | Str | 15 | T | A | 0 | 30 | 0 | 0.00000 | 1.00000 | 0.00000 | A2 |
| Ots_myo1a-384-36 | Str | 15 | C | T | 0 | 0 | 30 | – | – | 1.00000 | – |
| Ots_unk9480-51-38 | Str | 15 | C | T | 0 | 0 | 30 | – | – | 1.00000 | – |

Table S14. Parentage-based tagging results for 29 Coho Salmon misidentified in the lower Columbia River Chinook Salmon sport fishery of WA and OR, USA.

| **Individual** | **Sample Year** | **Adipose Absent** | **Parental Hatchery** | **Spawn Year** | **Inferred Age** | **FDR** | **LOD** |
| --- | --- | --- | --- | --- | --- | --- | --- |
| OtsZnlo21-02373 | 2021 | Yes | – | – | – | Inf | -10.91 |
| OtsZnlo21-02452 | 2021 | No | Dworshak National Fish Hatchery | 2018 | 3 | 0 | 36.78 |
| OtsZnlo21-02664 | 2021 | No | – | – | – | Inf | -5.37 |
| OtsZnlo21-02712 | 2021 | Yes | – | – | – | Inf | -13.78 |
| OtsZnlo21-02755 | 2021 | No | – | – | – | Inf | -11.18 |
| OtsZnlo21-02952 | 2021 | Yes | Bonneville Fish Hatchery | 2019 | 2 | 0.018 | 28.38 |
| OtsZnlo21-02975 | 2021 | Yes | – | – | – | Inf | -6.80 |
| OtsZnlo21-03251 | 2021 | Yes | – | – | – | Inf | -4.64 |
| OtsZnlo21-03980 | 2021 | Yes | – | – | – | Inf | -14.84 |
| OtsZnlo21-04192 | 2021 | Yes | – | – | – | Inf | -10.84 |
| OtsZnlo21-04332 | 2021 | Yes | – | – | – | – | – |
| OtsZnlo21-04892 | 2021 | No | – | – | – | – | – |
| OtsZnlo21-04907 | 2021 | Yes | Three Mile Dam Hatchery | 2018 | 3 | 0 | 36.95 |
| OtsZnlo21-04915 | 2021 | Yes | – | – | – | Inf | -14.03 |
| OtsZnlo21-05299 | 2021 | Yes | – | – | – | Inf | -5.20 |
| OtsZnlo21-05388 | 2021 | Yes | – | – | – | Inf | -3.96 |
| OtsZnlo22_00252 | 2022 | Yes | – | – | – | Inf | -6.96 |
| OtsZnlo22_00267 | 2022 | Yes | Eagle Creek National Fish Hatchery | 2019 | 3 | 0 | 35.00 |
| OtsZnlo22_05142 | 2022 | Yes | Eagle Creek National Fish Hatchery | 2019 | 3 | 1.00E-06 | 30.90 |
| OtsZnlo22_05150 | 2022 | Yes | – | – | – | Inf | -6.32 |
| OtsZnlo22_05155 | 2022 | Yes | Three Mile Dam Hatchery | 2019 | 3 | 0 | 33.89 |
| OtsZnlo22_05168 | 2022 | Yes | Eagle Creek National Fish Hatchery | 2019 | 3 | 0 | 33.12 |
| OtsZnlo22_07050 | 2022 | Yes | – | – | – | Inf | -14.32 |
| OtsZnlo22_07285 | 2022 | Yes | Eagle Creek National Fish Hatchery | 2019 | 3 | 0 | 36.60 |
| OtsZnlo22_07286 | 2022 | Yes | – | – | – | Inf | -13.89 |
| OtsZnlo22_07463 | 2022 | No | Bonneville Fish Hatchery | 2019 | 3 | 0.001 | 30.90 |
| OtsZnlo22_08455 | 2022 | No | – | – | – | 1 | -9.10 |
| OtsZnlo22_08871 | 2022 | No | – | – | – | Inf | -8.37 |
| OtsZnlo22_08872 | 2022 | Yes | Three Mile Dam Hatchery | 2019 | 3 | 0 | 35.06 |

Figure S1. Infographic describing the process of species scoring in *CallSpecies.py.*

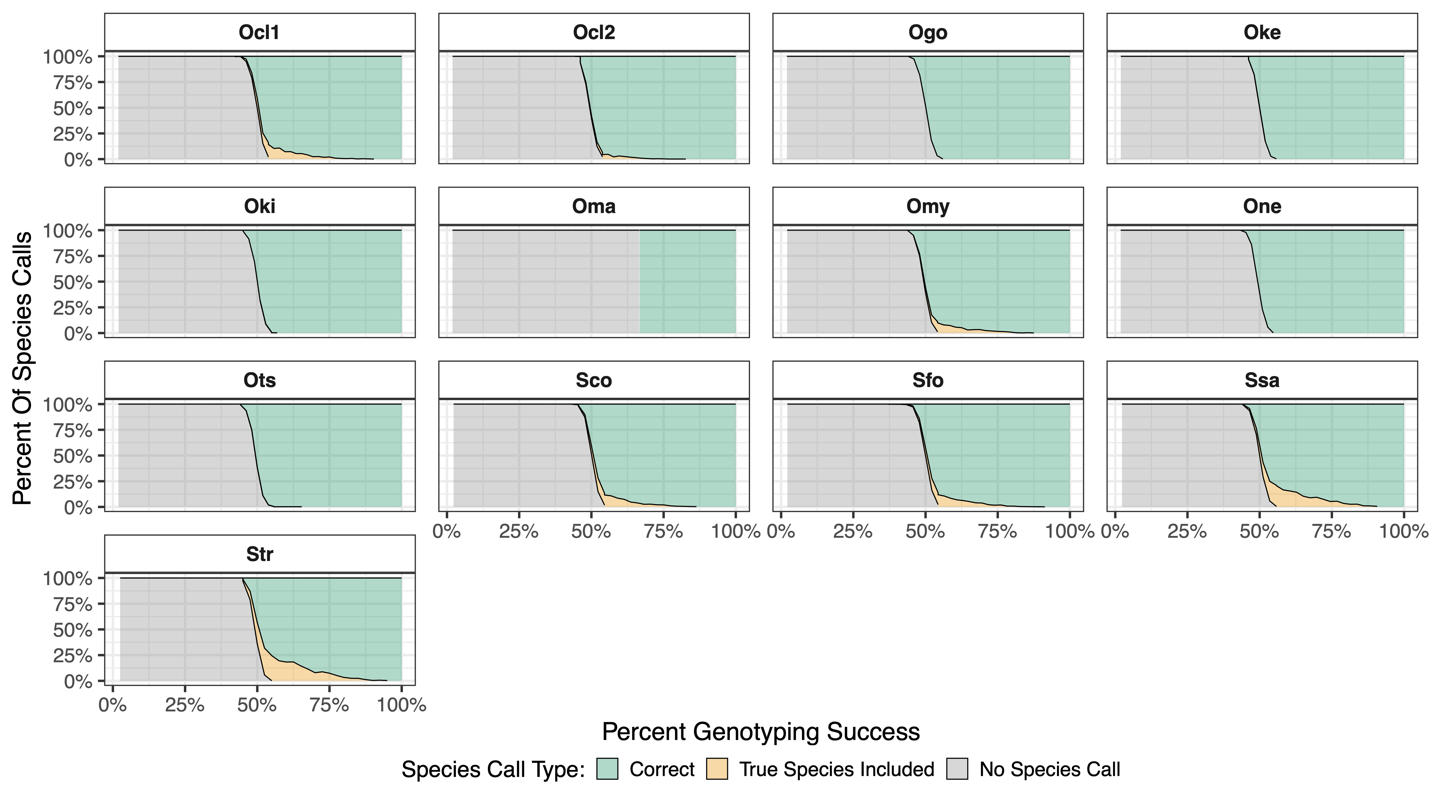


Figure S2. Effect of genotyping success of species-informative loci on species calling accuracy shown by species. The proportion of simulated genotypes for each species that resulted in an exclusive and correct species call are shown in green, the proportion that included the true species along with alternative species are shown in yellow, and simulated genotypes for which no species determination was made are shown in gray. Note that no species calls were made that did not include the correct species.

Figure S3. The effect of simulated shared polymorphisms among species on species calling accuracy. The percentage of species calls that are correct are reported for 1, 2, 5, 10, and 15 loci with shared polymorphisms at an allele frequency of 0.1, 0.05, 0.1, and 0.2. 95% bootstrapped confidence intervals are shown.

Figure S4. The effect of simulated shared polymorphisms among species on species calling accuracy. The percentage of species calls that include the true species with alternatives are reported for 1, 2, 5, 10, and 15 loci with shared polymorphisms at an allele frequency of 0.1, 0.05, 0.1, and 0.2. 95% bootstrapped confidence intervals are shown

Figure S5. The effect of simulated shared polymorphisms among species on species calling accuracy. The percentage of species calls that exclude the true species are reported for 1, 2, 5, 10, and 15 loci with shared polymorphisms at an allele frequency of 0.1, 0.05, 0.1, and 0.2. 95% bootstrapped confidence intervals are shown

Figure S6. The effect of simulated shared polymorphisms among species on species calling accuracy. The percentage of failures to make a species calls are reported for 1, 2, 5, 10, and 15 loci with shared polymorphisms at an allele frequency of 0.1, 0.05, 0.1, and 0.2. 95% bootstrapped confidence intervals are shown.

Appendix S1: Species Identification with existing GSI software.

Species identification using genetic population assignment software is a viable alternative to the numerical scoring approach employed in this manuscript. The benefits of our approach is that it minimizes dependencies ( Python V3+ and standard libraries), operates within a well-established genotyping pipelines without reformating genotypic input files, and uses a basic scoring rubric (species-seq file) that is easily modified by laboratory staff unfamiliar with statistical computing software. As a result, our approach achieved a key objective of delivering accurate genetic species identifications to laboratory technicians as standard genotyping pipeline output. However, for some researchers using a genetic stock idenification software for species identification may represent a trivial increase in time and effort, and may present some advantages. In this use case, representative genotypes of each species will be used as reference populations and unknown samples will be assigned to each population (i.e., species).

Importantly, not all genetic assignment software may work well for species identification with our marker panel. For example, older software programs often required that all loci atleast partially genotype in all reference populations (e.g., Piry et al., 2004). Additionally, when alleles were unshared among reference populations, as is the case with diagnostic species loci, user-defined values were often assigned to minor allele frequences (e.g, 0.01) to permit probability calculations. Any selected software would need to be tested to ensure it is adequate for species identification under a variety of conditions. However, modern Bayesian or partially Bayesain approaches such as those implemented in the R-package *Rubias* (Moran & Anderson, 2019) circumvent these potential issues and appear to perform quite well. For demonstration purposes, we applied a genetic stock identifiaction approach using *Rubias* to our two simulated datasets testing missing data and shared polymorphisms presented in the main text. The reference populations were comprised of 1000 representative genotypes from each species. The simulated individuals were from each simulation scenario were randomly assigned to 50 mixture collections (unknown species samples). We then executed the *infer_mixture* function using default settings (MCMC method, 2000 estimation iterations, and 100 burn-in). The species calls were obtained by taking the maximum posterier probability of an unknown sample being from each reference population (species), which is reported as “PofZ” in the output. Please note that these results should not be considered a direct comparison of our numerical scoring method to *Rubias*, because user-defined thresholds could certianly be employed to improve the accuracy of species inference by Rubias. For example, one could use a PofZ threshold (e.g., >0.5) to make a species call versus no call, or a use a threshold that allows for approximate ties and call two or more species jointly similairly to our numerical scoring method. Additional optimization of Rubias settings may also improve inference.

The species assignment results obtained from Rubias for simulations of missing genotypic data and shared polymorphisms demonstrate that species calls based upon the species with the highest posterior probability are highly accurate under most reasonable conditions (Figure S7 and S8). Although our numerical scoring approach makes less errors (exclusion of the True Species) with default thresholds (see main text), establishing user-defined assignment thresholds for Rubias would likely achieve similar species identification accuracies. However, for reference purposes, CallSpecies.py had the following accuracy and error rates when simulating 15 loci with shared polymorhpisms at a minor allele frequency of 0.2 : 99.25% exclusively correct to the simulated species, 0.38% with true species included among alternatives, 0.38% true species excluded (incorrect), and 0% with no species calls. This is compared to Rubias which was 94.81% exclusively correct to the simulated species when calling the species with the highest posterior probability of membership in the same scenario (Figure S8). Beyond the practical advantages of our method previously outlined, the excution time for Rubias’s *infer_mixture* function was 136.25s compared to 67.42s for CallSpecies.py (~2x faster) on the simulation of missing genotypic data containing 586,000 multilocus individual genotypes using a 2021 MacBook Pro M1 (Model Number: MK183LL/A). Notably, the reported execution time for Rubias does not include reformatting input files, post-processing and making species determinations, or I/O operations.

Literature Cited:

Moran, B. M., & Anderson, E. C. (2019). Bayesian inference from the conditional genetic stock identification model. *Canadian Journal of Fisheries and Aquatic Sciences*, *76*(4), 551–560. https://doi.org/10.1139/CJFAS-2018-0016/SUPPL_FILE/CJFAS-2018-0016SUPPLA.PDF

Piry, S., Alapetite, A., Cornuet, J.-M., Paetkau, D., Baudouin, L., & Estoup, A. (2004). GENECLASS2: A Software for Genetic Assignment and First-Generation Migrant Detection. *Journal of Heredity*, *95*(6), 536–539. https://doi.org/10.1093/jhered/esh074


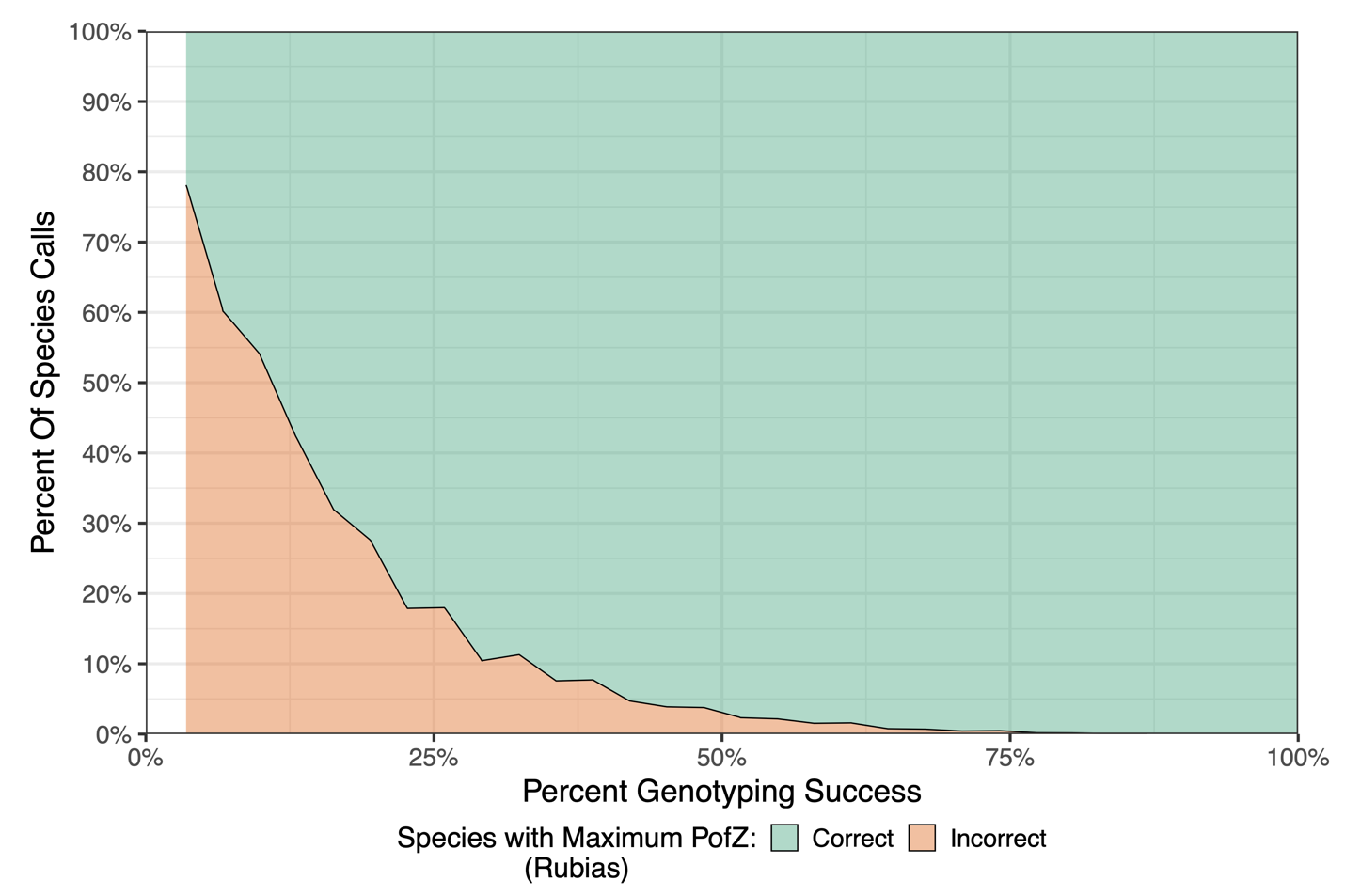


Figure S7. Effect of genotyping success of species-informative loci on species calling accuracy using the R-package *Rubias*. Species were called based on the species with the highest posterior probability (i.e., maximum PofZ). Species calls that correspond to the simulated species are shown in green (correct) and those that are discordant (incorrect) are shown in orange. All levels of missing data are shown {*n*_loci_..1}.

Figure S8: The effect of simulated shared polymorphisms on species calling accuracy using the R-package *Rubias*. Species were called based on the species with the highest posterior probability (i.e., maximum PofZ). The percentage of species calls that are correct are reported for 1,2,5,10, and 15 loci with shared polymorphisms at an allele frequency of 0.1, 0.05, 0.1, and 0.2. The percentage of correct calls reflects an average among all species considered, excluding Masu Salmon. 95% bootstrapped confidence intervals are shown.
